# A Yolk@Shell Photothermal Structure for Integrated Solar‐Driven Undrinkable Water Purification and Thermoelectric Power Generation

**DOI:** 10.1002/advs.202523455

**Published:** 2026-01-25

**Authors:** Minrui Zhan, Xiang Fu, Yumei He, Manman Zhao, Xi Zhang, Rong Liu, Haonan Yang, Ming Yang, Huiyu Yang, Pei Lyu, Jiehao Du, Shaojin Gu, Xin Liu, Bin Shang

**Affiliations:** ^1^ State Key Laboratory of New Textile Materials and Advanced Processing School of Materials Science and Engineering Wuhan Textile University Wuhan P. R. China; ^2^ School of Chemistry and Materials Science Hubei Engineering University Xiaogan P. R. China; ^3^ Hubei Integrative Technology and Innovation Center for Advanced Fiberous Materials Wuhan P. R. China

**Keywords:** porous silicone sponge, solar‐driven interfacial vapor evaporation, thermoelectric generation, wastewater purification, yolk@shell structure

## Abstract

Integrated solar‐driven vapor generation and thermoelectric power generation have been considered a promising solution to alleviate the global freshwater shortage and energy crisis. However, the long‐standing trade‐off between high‐speed water evaporation and efficient thermoelectric generation remains a challenge. In this study, a yolk@shell structured photothermal evaporator (denoted as HSS@MNPs) composed of super‐elastic hydrophobic silicone sponge, super‐hydrophilic epoxy resin and photothermal melanin nanoparticles was prepared via simple sol‐gel and spray coating methods. The results show that various waste liquids can be effectively purified, and a series of experiments and analyses demonstrate that the height of the HSS@MNPs and the position of the thermoelectric (TE) module within the photothermal structure affect its water evaporation and thermoelectric performance. By taking advantage of the unique yolk@shell structure of HSS@MNPs, the position of the TE module can be optimized without destroying the self‐floating property, thermal insulation, photothermal property, and water transmission performance of the evaporator. This results in efficient and stable evaporation (3.08–3.17 kg m^−2^ h^−1^)‐thermoelectric (135.4–144.6 mV) co‐generation under one sun irradiation. The outdoor application was also demonstrated, providing a straightforward strategy to resolve the long‐standing trade‐off between high‐speed water evaporation and efficient thermoelectric generation.

## Graphical abstract

A yolk@shell structured photothermal evaporator was prepared via simple sol‐gel and spray coating methods. By taking advantage of the unique yolk@shell structure, the position of the TE module within the evaporator can be optimized without affects its water evaporation performance. This results in efficient and stable evaporation (3.08‐3.17 kg m^−2^ h^−1^)‐thermoelectric (135.4‐144.6 mV) co‐generation under one sun irradiation, providing a straightforward strategy to resolve the long‐standing trade‐off between high‐speed water evaporation and efficient thermoelectric generation.

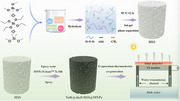

## 1 Introduction

Solar energy, as a green energy, is characterized by its inexhaustible nature and has been widely applied in fields such as energy storage [1], catalysis [2, 3], atmosphere water harvesting [4, 5, 6], wastewater treatment [7, 8, 9], etc. Among these applications, the combination of solar‐driven interfacial water evaporation technology and thermoelectric technology can effectively address two significant global challenges caused by the increasing population and industrialization: clean water and energy.

Different from the traditional bulk heating technology which requires heating the entire water body to promote water evaporation, the interfacial heating technology is more effective because it only needs to transport water to the air/water interface and utilize the energy generated at the interface to heat the local water [10, 11]. Therefore, for a high‐performance evaporator, it not only needs to have the basic self‐floating property but also should possess excellent thermal management and water supply capabilities. To date, a wide variety of photothermal substances [12, 13, 14, 15, 16] and porous structures [17, 18, 19, 20, 21] have been developed. Through enhancing solar absorption [22, 23, 24, 25], reducing the water evaporation enthalpy [26, 27, 28, 29], and optimizing water supply and heat loss [30, 31, 32], the evaporation performance of the evaporator has been significantly improved.

To enable concurrent water evaporation and thermoelectric power generation, multiple approaches have been developed that combine interfacial photothermal technology with the Seebeck effect [33, 34, 35, 36, 37, 38, 39, 40]. For example, inspired by mangrove, Ji et al., fabricated a TiN/TiO_2_@carbon cloth (CC) nanotubes array, it can achieve consistent evaporation rates of 2.02 kg m^−2^ h^−1^ and attain a maximum thermoelectricity output of 261.4 mV under 1 sun irradiation [34]. Zheng et al., reported a fully biomass‐based bilayer multifunctional solar evaporator that consists of an upper lignin‐derived porous carbon (LPC)‐embedded chitosan/lignin (CSL) composite aerogel layer featuring vertically small channels and an underlying hydrophilic CS aerogel. The obtained bilayered aerogel evaporator achieved a water evaporation rate of 1.717 kg m^−2^ h^−1^ under1 kW m^−2^ irradiation and generated a voltage output of 279 mV under 3 kW m^−2^ irradiation, respectively [37]. Integrating thermoelectric (TE) modules with 2D or 3D evaporators and arranging them at the evaporator bottom is a common system integration strategy for combined water and power generation. According to the thermoelectric energy generation mechanism based on the Seebeck effect, it is attributed to the temperature difference between the hot side and the cold side of the TE module. Under solar illumination, the heat produced by the evaporator via photothermal conversion is unevenly distributed, primarily concentrating on the illuminated surface. Typically, placing the TE module beneath a 2D evaporator facilitates a greater temperature gradient between the hot and cold sides, thereby enhancing the output voltage. However, 2D evaporators exhibit limited thermal management capacity, often leading to suboptimal evaporation performance. In previous studies, only a limited number of works have achieved efficient steam generation by integrating water film and cluster evaporation strategies [41]. Conversely, 3D evaporators offer superior thermal management, enabling more effective heat concentration at the evaporation interface and thus better evaporation performance. Nevertheless, due to challenges in heat transfer to the bottom region, the temperature difference across the TE remains modest, which constrains the overall electrical energy output. Although some researches have proposed to balance evaporation and thermoelectric performance through layout adjustments of TE within a 3D evaporator, several critical issues are frequently neglected. First, embedding the module compromises structural integrity, markedly impairing water transport capacity of evaporator. Second, as the system operates, inadequate heat dissipation at the cold end of the module can lead to persistent temperature accumulation in this region. Therefore, the development of an efficient evaporator that not only exhibits outstanding water evaporation performance—such as self‐floating ability, excellent photothermal properties, and effective water management—but also allows flexible adjustment of the position of internal thermoelectric components remains a key research priority.

Herein, a yolk@shell structure with a hydrophobic and heat‐insulating yolk and super‐hydrophilic and light‐absorbing shell was successfully fabricated via simple sol‐gel and spray methods. The photothermal component employed in this evaporator consists of melanin particles. Although typically considered a waste product, these particles exhibit exceptional light absorption properties via nonradiative relaxation of rich delocalized π electrons [42]. The hydrophobic and heat‐insulating sponge can be fabricated via a straightforward one‐step hydrolytic condensation process, which is simple to execute and requires minimal operational complexity. Moreover, benefiting from its unique yolk@shell structure, the position of the TE module can be flexibly adjusted without significantly destroying the self‐floating property, thermal insulation, photothermal property, and water transmission performance of the evaporator. Therefore, by further optimizing the distribution of heat and water in the evaporator, the long‐standing trade‐off between high‐speed water evaporation and efficient thermoelectric generation is expected to be resolved, and it promises to achieve efficient evaporation‐thermoelectric co‐generation.

## 2 Results and Discussion

**FIGURE 1:**
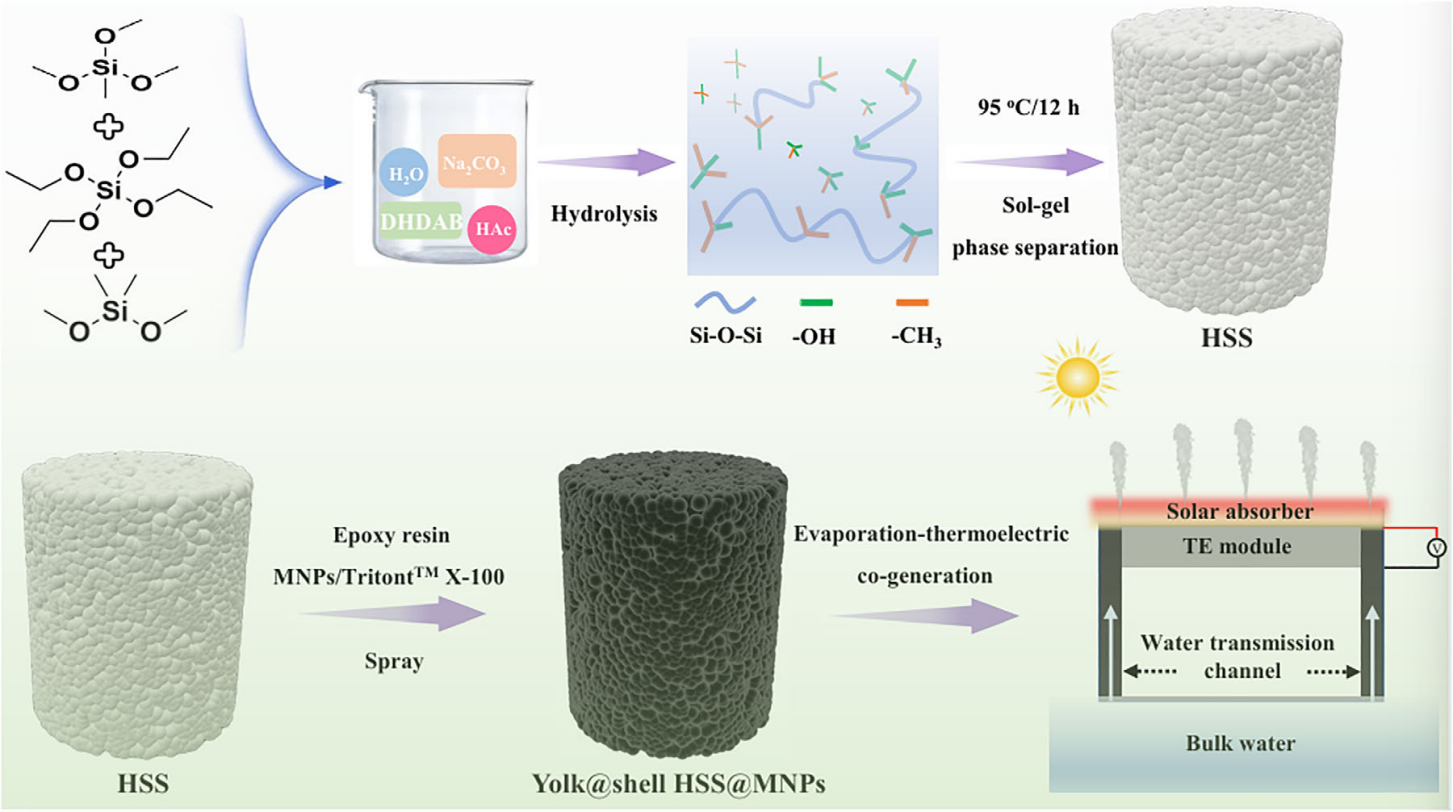
Preparation diagram of the proposed yolk@shell HSS@MNPs.

The proposed yolk@shell photothermal structure (denoted as HSS@MNPs) was fabricated according to Figure 1. Initially, through the hydrolytic condensation of TEOS, MTMS and DMDMS, hydrophobic silicone sponges (denoted as HSS) of different heights, sizes or shapes can be obtained when changing the molds used (Figure S1). Subsequently, the prepared sponge is used as the substrate, and a solution containing melanin particles (MNPs), Triton^TM^ X‐100 and epoxy resin is sprayed onto it for super‐hydrophilic modification. The thickness of the sponge surface can be modified by controlling the solution composition and spraying conditions, thereby preparing the yolk@shell structured photothermal sponge. After successfully completing the above synthesis steps, the obtained sponge is expected to simultaneously possess super‐hydrophilic water transport channels, excellent thermal insulation, self‐floating and photothermal properties. Further controlling the height of the photothermal structure and the position of the TE module can regulate the distribution of heat and water in it, which is conducive to achieving efficient purification of wastewater driven by sunlight and the simultaneous production of electricity.

**FIGURE 2:**
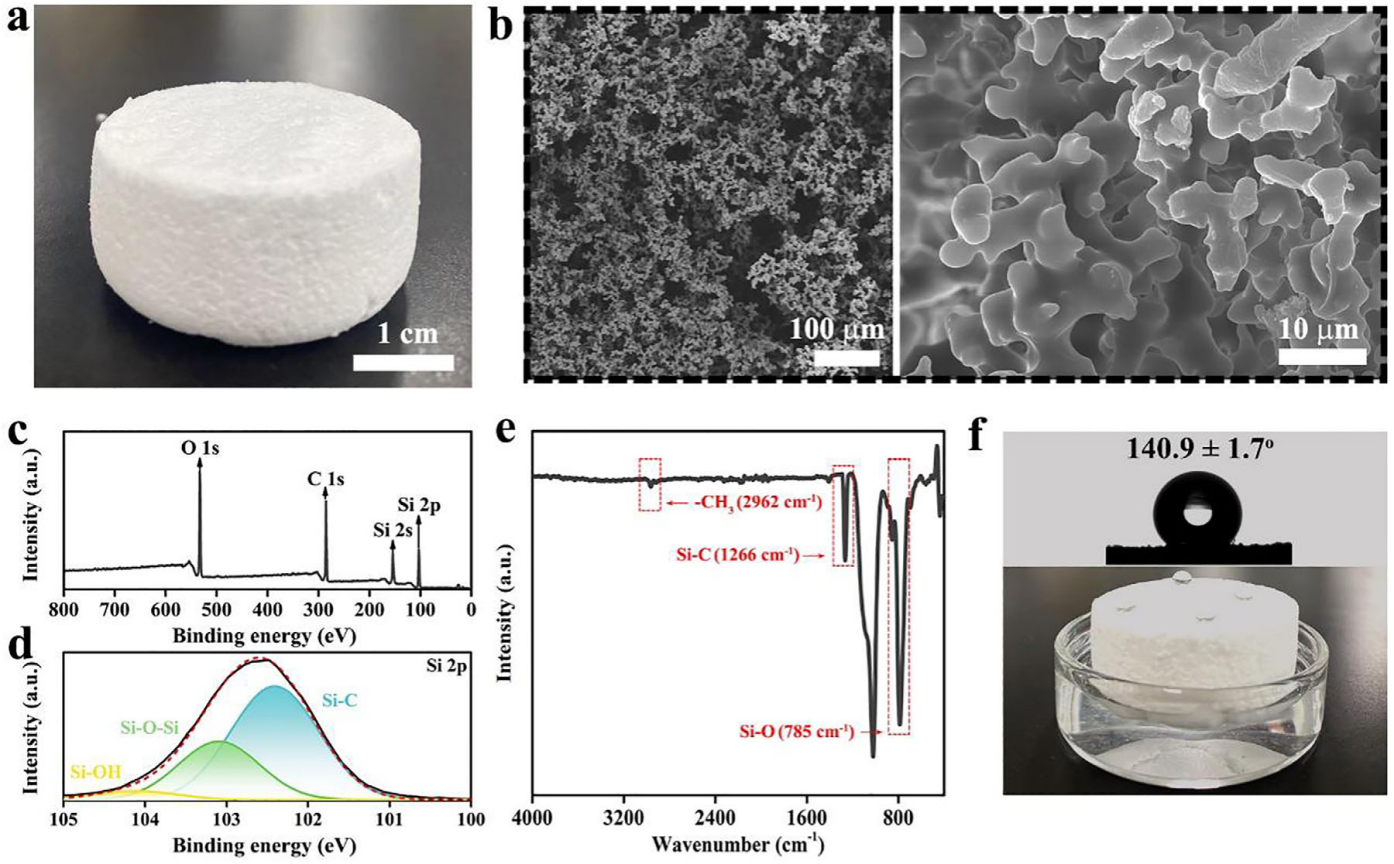
Photograph (a), SEM images (b), XPS spectrums (c,d), FTIR spectrum (e) and hydrophobicity test (f) of the fabricated HSS.

Figure 2a presents the optical photograph of the fabricated HSS. The gelation reaction between TEOS, MTMS, and DMDMS yields a white‐colored, porous (with an average pore diameter of approximately 17 nm and a BET surface area of about 0.87 m^2^ g^−1^, as shown in Figure S2) and super‐elastic structure (Figure 2b). The porous structure has an extremely low thermal conductivity (the thermal conductivity is approximately 0.044 W m^−1^ K^−1^), and demonstrates rapid recovery and retention of structural integrity even after undergoing multiple compression cycles at varying strain levels of 20% and 50% respectively (Figure S3). In the X‐ray photoelectron spectrum (XPS) of the prepared HSS (Figure 2c), the Si 2p, Si 2s, C 1s, and O 1s peaks appeared. According to the Si 2p XPS spectrum (Figure 2d), the HSS is formed mainly by Si─C and Si─O─Si bonds with a few Si─OH groups. With temperature increases, the molecular structure of HSS remains stable, with no significant thermal weight loss observed. A noticeable mass reduction occurs because of the oxidation and/or decomposition of methyl groups only when the temperature surpasses 350°C (Figure S4). Due to the presence of a large number of hydrophobic groups (─CH_3_) in the gel network structure (Figure 2e), the HSS with a water contact angle of approximately 140.9° ± 1.7° can stably float on the water without being wetted (Figure 2f).

**FIGURE 3:**
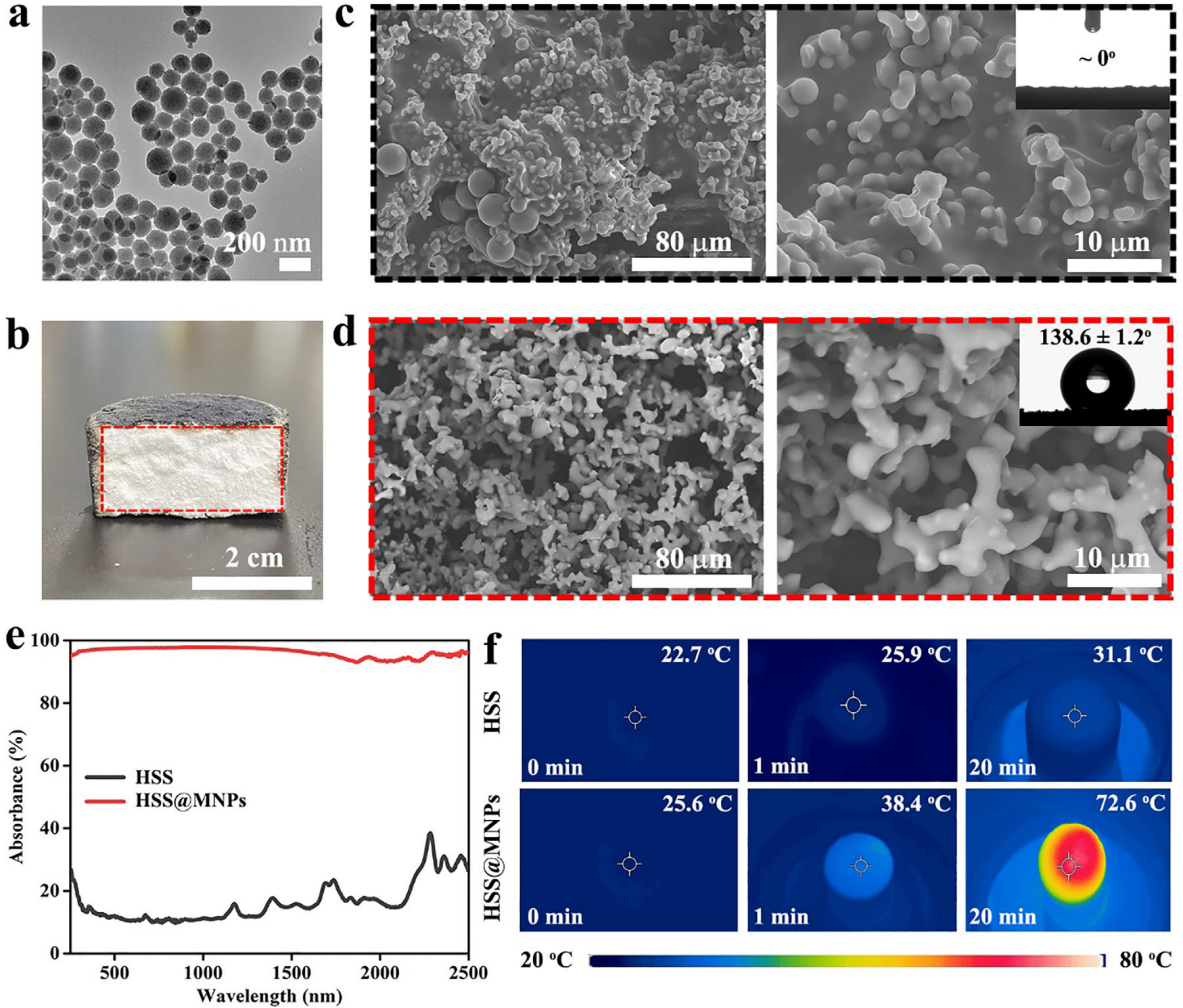
(a) TEM image of MNPs. (b) Cross‐sectional photograph of the fabricated yolk@shell HSS@MNPs. SEM images of the shell (c) and yolk (d) of the fabricated yolk@shell HSS@MNPs, and corresponding picture of water contact angle. UV–vis–NIR absorption spectra (e) and corresponding infrared images (f) of HSS and HSS@MNPs.

Conversely, after modifying MNPs (approximately 187 nm in size, as shown in Figure 3a; Figure S5) and hydrophilic resin (Triton ^TM^ X‐100 and epoxy resin) on the sponge surface via a simple spray coating method, the sponge appears black (Figure 3b). The rough surface of the sponge is covered with a layer of coating, and the wettability transforms from hydrophobic to super‐hydrophilic (Figure 3c). When water droplets come into contact with it, they can rapidly wet the surface of the material, thus providing a feasible channel for the transmission of water on the sponge surface. Notably, compared to that before spraying, the internal color, structure, and wettability of the aerogel did not change significantly (Figure 3d). This is because the high viscosity of the spray liquid and its rapid cross‐linking properties prevent it from effectively penetrating into the interior of the sponge, only allowing for surface modification to a limited depth.

Strong light absorption across the entire solar spectrum is crucial for achieving efficient photothermal water evaporation. The optical properties of the HSS and HSS@MNPs were precisely characterized using ultraviolet‐visible‐near‐infrared (UV–vis–NIR) spectroscopy. Compared with the poor solar absorbance capacity of the HSS, the fabricated HSS@MNPs exhibits a stronger ability to capture sunlight due to the modification of light‐absorbing MNPs (Figure S6). Specifically, within the 250–2500 nm range, the average solar absorbance of HSS and HSS@MNPs is approximately 16% and 96%, respectively (Figure 3e). After being exposed to simulated sunlight of 1 kW m^−2^ for 20 min, the surface temperature of HSS@MNPs quickly increased and finally reached around 72.6°C during multiple cycle tests (Figure 3f; Figure S7), with a temperature increase of approximately 47°C. However, under the same test conditions, the surface temperature of HSS only increased from 22.7°C to 31.1°C, reflecting the poor photothermal conversion capacity.

**FIGURE 4:**
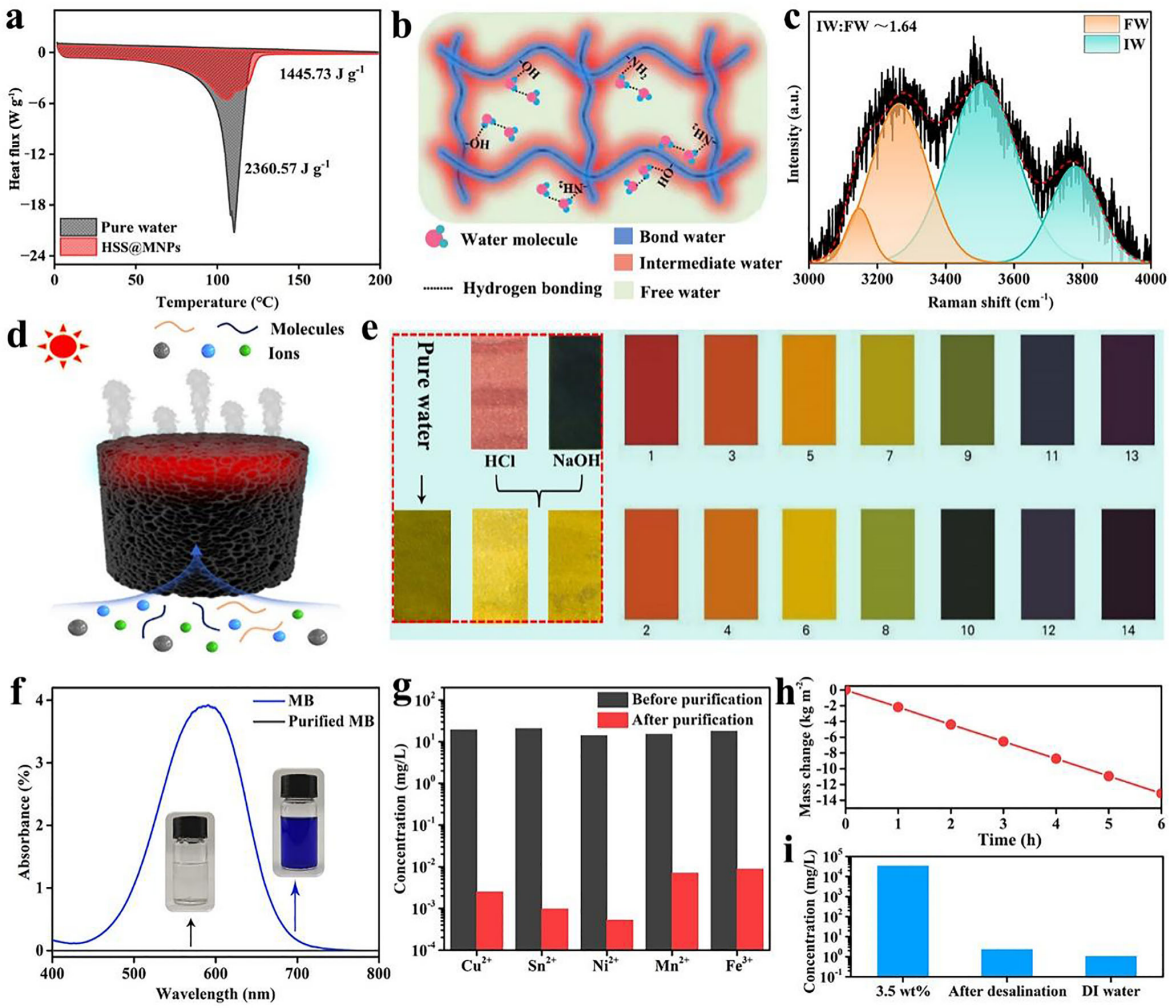
(a) The enthalpy of evaporation of pure water and HSS@MNPs. (b) Schematic of water in the hydratable polymer network of the shell of HSS@MNPs. (c) Raman spectroscopic characterization of different water states in HSS@MNPs. (d) Schematic diagram of water purification via solar vapor generation. (e) Comparison of pH values of acidic solution and alkaline solution before and after purification. (f) The ultraviolet absorption spectrum and photos of methyl blue dye wastewater before and after purification. (g) Ion concentration of heavy metal wastewater before and after purification. (h) The curve of the change in the mass of 3.5 wt.% NaCl solution over time when HSS@MNPs is used as evaporator. (i) Na^+^ concentration in the 3.5 wt.% NaCl solution before and after purification, along with the Na^+^ level in the DI water.

Owing to its low thermal conductivity, super‐hydrophilic surface, and remarkable photothermal conversion efficiency, the HSS@MNPs (a cylindrical structure measuring 2 cm in height and 6 cm in diameter) demonstrates remarkable evaporation performance when used as an evaporator. It enables the rapid evaporation of various common wastewaters, including acidic/alkaline solutions, dye effluents, and heavy metal wastewater, with high evaporation rates of approximately 2.21, 2.23, 2.21, and 2.27 kg m^−2^ h^−1^ respectively (Figure S8). The rapid evaporation of water can be attributed to the reduced evaporation enthalpies (Figure 4a). Typically, both MNPs and Triton ^TM^ X‐100 are rich in hydrophilic groups (─OH, ─NH_2_), these hydrophilic groups can form strong hydrogen bonds with water molecules (bound water), partially activating the water (increase intermediate water content) and reduce the amount of energy required for evaporation (Figure 4b) [43]. To elucidate the mechanism of enhancing solar evaporation over HSS@MNPs, water states in it were analyzed using Raman spectra. The calculated molar ratio of intermediate water (IW): free water (FW) in HSS@MNPs was 1.64:1 (Figure 4c). These results indicate that most of the water contained in HSS@MNPs is intermediate water, this reduces the evaporation enthalpy of water and accelerates solar vapor generation.

By converting liquid water into vapors via the photothermal method, impurities in water can be separated from water molecules, enabling the separation and purification of non‐potable water (Figure 4d). As shown in Figure 4e, when the HSS@MNPs was placed in an acidic solution (pH∼*1*) and an alkaline solution (pH∼*13*) to accelerate water evaporation under 1 kW m^−2^ solar irradiation, the acidity and alkalinity of the water collected by re‐condensation were both close to seven, which is similar to the pure water we used. Additionally, after purification, the dye wastewater became transparent, and the characteristic absorption peak of methyl blue dye molecules could not be detected in the ultraviolet spectrum (Figure 4f); the concentrations of Cu^2+^, Sn^2+^, Ni^2+^, Mn^2+^ and Cu^3+^ in the heavy metal wastewater decreased significantly from 19.2, 20.8, 14, 15.2, and 18 mg L^−1^ to 2.5, 0.97, 0.52, 6.9, and 8.8 ug L^−1^ (Figure 4g). The ion removal rates all exceeded 99.9% (Figure S9). Even after three days of continuous immersion in the aforementioned waste liquids, the HSS@MNPs exhibited no significant reduction in its evaporation enhancement performance (Figure S10). Moreover, for a 3.5 wt.% NaCl solution, the mass change exhibited a linear relationship with time over a 6 h test period, indicating a consistent evaporation rate that was unaffected by variations in salt ion concentration (Figure 4h). Following photothermal purification, the sodium chloride salinity was significantly reduced (Figure 4i).

**FIGURE 5:**
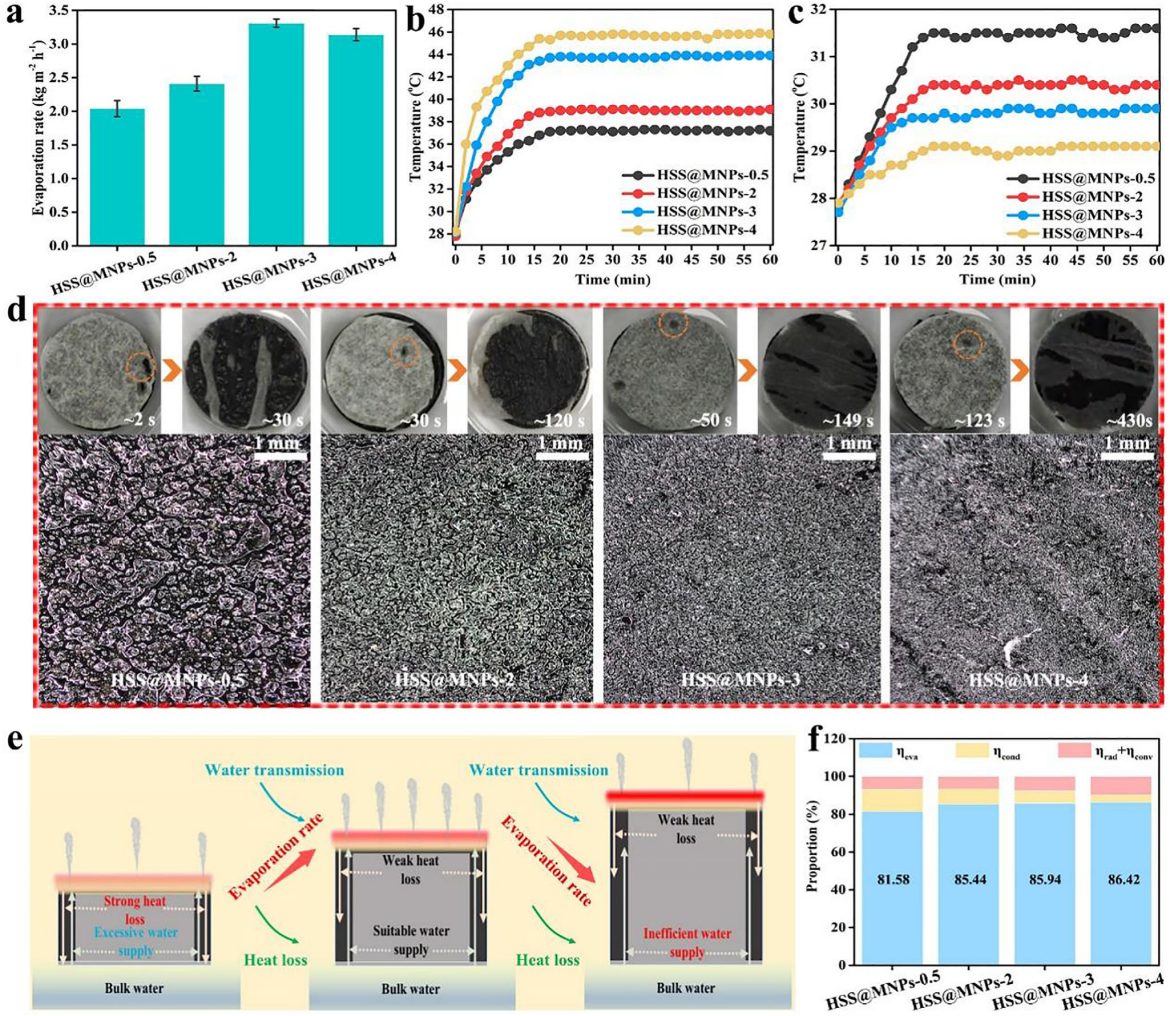
(a) Evaporation rates of pure water when using HSS@MNPs with different height as an evaporator. (b) Surface temperature variation curve of different evaporators. (c) Temperature variation curves of water at the bottom of different evaporators. (d) Comparison of water transport capacity of different evaporators and optical images of surface water distribution. (e) Schematic diagram of water supply and interfacial heating of the solar evaporator. (f) Various energy losses of different evaporators during the evaporation process.

In order to investigate the influence of evaporator height on water evaporation performance, another three cylindrical evaporators (the diameter is approximately 6 cm) with heights of 0.5, 3, 4 cm, respectively were prepared. These evaporators are denoted as HSS@MNPs‐0.5, HSS@MNPs‐2, HSS@MNPs‐3, and HSS@MNPs‐4. As summarized in Figure 5a, when the height of the evaporator rises, the calculated evaporation rate exhibits a tendency of first increasing and then decreasing. Among them, HSS@MNPs‐3 has the fastest water evaporation rate of approximately 3.31 kg m^−2^ h^−1^. Neither too high nor too low an evaporator height can achieve the optimal performance. It is well known that for solar‐driven interfacial vapor generation technology, both the thermal localization and water management of the evaporation surface are closely related to the evaporation performance. In Figure 5b, the HSS@MNPs‐4 surface reaches the highest equilibrium temperature of approximately 45.8 °C under solar irradiation (1 kW m^−2^), followed by HSS@MNPs‐3 and HSS@MNPs‐2, while HSS@MNPs‐0.5 shows the lowest thermal equilibrium temperature of approximately 37.2°C. Interestingly, for the evaporator with the highest surface temperature, the temperature change of the bottom water is the smallest (Figure 5c). Additionally, it takes approximately 2, 30, 50, and 123 s, respectively, for water to be transported from the bottom to the top of the HSS@MNPs‐0.5, HSS@MNPs‐2, HSS@MNPs‐3, and HSS@MNPs‐4, while it takes approximately 30, 120, 149, and 430 s respectively, to fully wet their surfaces. An increase in evaporator height leads to a longer transport time of water to the evaporation surface, and reduces water content at the surface (Figure 5d).

The different water transport capabilities of these evaporators can be analyzed from the perspective of capillary forces. Generally, the capillary action generated between the super‐hydrophilic shell and water prompts the water to overcome gravity and move upward. However, as the transmission height increases, the capillary action weakens, restricting the stable supply of water. To achieve rapid water evaporation, it is crucial to provide a stable water supply to the evaporation surface, and reducing the height of the evaporator can effectively accelerate the water delivery speed to the evaporation surface. If not properly managed, such regulation may result in excessive water supply on the evaporation surface. This, on the one hand, reduces the utilization efficiency of heat; on the other hand, the water flowing back will transfer a large amount of heat generated at the photothermal interface to the bottom water, raising the temperature of the bottom water and causing heat waste, as illustrated in Figure 5e. From an energy loss perspective (see Supporting Information), HSS@MNPs‐4, HSS@MNPs‐3 and HSS@MNPs‐2 exhibit almost similar high evaporation efficiencies, at 86.42%, 85.94%, and 85.44% respectively, whereas that of HSS@MNPs‐0.5 is slightly lower, at 81.58% (Figure 5f). When the evaporator height is low, heat conduction contributes more significantly to energy loss. Although increasing the evaporator height helps confine heat to the upper region—thus reducing downward conductive losses—it also exacerbates energy dissipation through radiation and convection. If the evaporation efficiency is calculated directly using Formula (10‐12) (see Supporting Information), the values for HSS@MNPs‐0.5, HSS@MNPs‐2, HSS@MNPs‐3, and HSS@MNPs‐4 are determined to be 84.5%, 99.9%, 138.9%, and 132.5%, respectively (Figure S11). The evaporation efficiency calculated from the evaporation rate is higher than that obtained from energy loss analysis. These elevated values can be attributed to unavoidable side evaporation from the materials, which substantially enhances the actual evaporation rate [44]. Therefore, an effective strategy for achieving high evaporation efficiency is to moderately increase the evaporator height to minimize conductive loss, while avoiding excessive radiation and convection losses.

**FIGURE 6:**
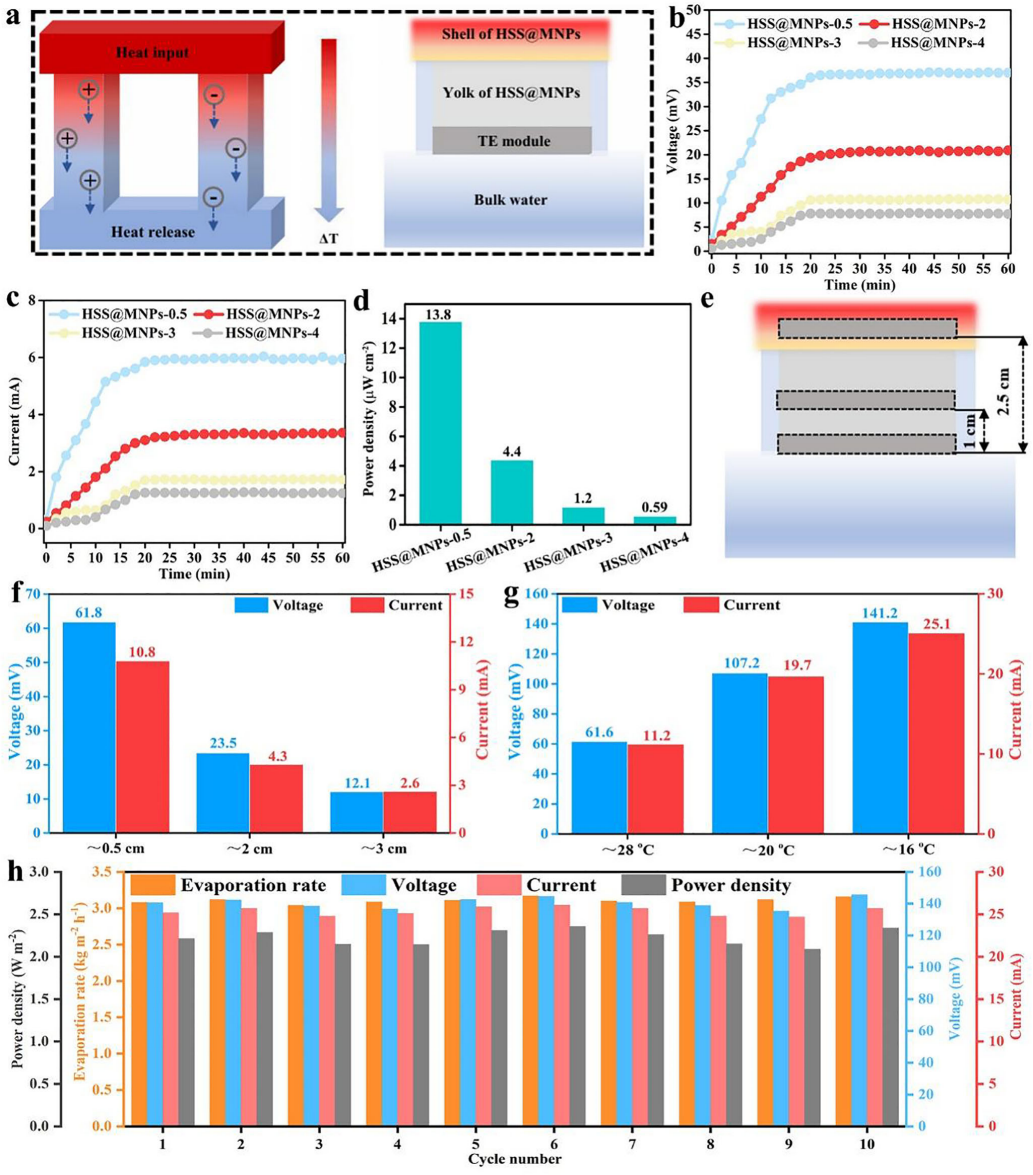
(a) Thermoelectric generation mechanism of the Seebeck effect. Voltage (b) current (c) and power density (d) generated by the module when placed at the bottom of different evaporators. (e) Schematic diagram of the adjustment module in the HSS@MNPs‐3. (f) The generated maximum output voltage and current of the module when it is placed at different positions in the HSS@MNPs‐3. (g) The generated maximum output voltage and current of the module when the cold end of the module is approximately 0.5 cm away from the top of the HSS@MNPs‐3 under different test environments. (h) Long‐term stability test.

During water evaporation, a temperature difference is formed along the longitudinal direction of the evaporator. When the TE module is embedded in the evaporator, the temperature difference formed between the hot end and the cold end of the module enables the system to generate TE power based on the Seebeck effect (Figure 6a). Usually, the TE module is placed at the bottom of the evaporator, and the thermoelectric performance is tested under solar irradiation (the light intensity, ambient temperature and relative humidity are approximately 1 kW m^−2^, 28°C, and 50%, respectively). In Figure 6b,c, the output voltages and currents vary significantly across different evaporator bottom modules. Nevertheless, at the start of the test, all modules exhibit a rapid increase in both voltage and current outputs before stabilizing and remaining essentially constant. Specifically, the output voltage decreases gradually from 37 mV for HSS@MNPs‐0.5 to 7.7 mV for HSS@MNPs‐4 as the evaporator height increases, while the corresponding output current declines from 5.96 to 1.23 mA. Both the output voltage and current of the evaporator are inversely proportional to its height. As shown in Figure 6d, the maximum output power density for HSS@MNPs‐0.5, HSS@MNPs‐2, HSS@MNPs‐3, and HSS@MNPs‐4 are 13.8, 4.4, 1.2, and 0.59 µW cm^−2^, respectively. According to the thermoelectric generation mechanism of the Seebeck effect, a temperature difference between the hot end and the cold end of a thermoelectric module can generate an electric current. For HSS@MNPs‐0.5, the fast water backflow and lower height are conducive to the water supply and heat conduction from the photothermal surface to the TE module (Figures S12 and S13). Although a significant change in the temperature of the cold end in HSS@MNPs‐0.5 was detected compared to that of other evaporators, the temperature difference between the two ends of TE module in HSS@MNPs‐0.5 was still the largest among all the samples (Figure S14). In contrast, when the height of evaporator increase, more heat is generated at the air/water interface but less heat can be transferred downward, causing the temperature difference between the hot side and the cold side of the TE module to be very small, which is not conducive to generating TE power.

In this study, the evaporator has a yolk@shell structure, in which the super‐hydrophilic shell captures sunlight and serves as a water transport channel, while the hydrophobic yolk provides stable self‐floating and heat insulation properties. By adjusting the position of the TE module in the evaporator as shown in Figure 6e, it is expected to enhance its photoelectric performance without significantly affecting the original evaporation performance. Consequently, HSS@MNPs‐3, which exhibited the best evaporation performance, was chosen to further optimize its thermoelectric performance. Results in Figures S15 and S16 indicate that when the TE module is placed at the bottom of the evaporator, the temperature of both the hot side and the cold side are relatively low, with a temperature difference of only 1.4°C. As the module rises in the evaporator (the distances from the cold ends of the modules to the top of the HSS@MNPs‐3 are approximately 2 and 0.5 cm respectively), it gradually approaches the photothermal interface, causing a significant increase in the temperature of its hot side. Notably, since the cold side of the module is always in contact with the air and can transfer the heat into water through air, the temperature of its cold side remains relatively stable. Therefore, as the module keeps approaching the evaporation interface, the temperature difference between the hot end and the cold end gradually increases (Figure S17), and the corresponding maximum output voltage and current reaches up to 61.8 mV and 10.8 mA, respectively (Figure 6f).

Interestingly, it was observed that the photothermal performance of the evaporator did not decrease significantly even when the ambient temperature was reduced to approximately 20 °C and 16 °C (Figure S18). Meanwhile, the temperature at the cold end of the module is almost the same as the ambient temperature, which is at a relatively low level (Figure S19). At low ambient temperature, the thermoelectric module generated a larger temperature difference between the hot end and the cold end (Figure S20), and its thermoelectric performance is often more outstanding. As presented in Figure 6g, under ambient conditions of approximately 16 °C, this module is capable of delivering maximum outputs of up to 141.2 mV and 25.1 mA. To investigate the evaporation ‐thermoelectric co‐generation of the proposed evaporator, the evaporation and thermoelectric generation performance of HSS@MNPs‐3 (the distance from the cold ends of the modules to the top of the HSS@MNPs‐3 is approximately 0.5 cm) were tested at approximately 16°C. During multiple cycle tests, the system's evaporation rate was stably maintained within the range of 3.04 to 3.17 kg m^−2^ h^−1^, with an output voltage of 135.4 to 144.6 mV and an output current of 24.7 to 26.1 mA, and the maximum power density could reach 2.09 to 2.34 W m^−2^. The overall operation stability was good (Figure 6h). Notably, due to the non‐destructive integration design with the external evaporator, the thermoelectric module has high layout flexibility and can be placed close to the photothermal surface to enhance the thermal input efficiency. Meanwhile, the heat accumulated at the cold end of the module can be transferred to the water at the bottom through the air below, ensuring the continuous stability of the thermoelectric performance. In contrast, if the module is directly inserted into the evaporator at the same height but retains its “yolk” structure below, the cold end temperature will gradually increase with the test time (Figure S21), with a significant decrease in output voltage from 125 to 88 mV (Figure S22). Consequently, the proposed HSS@MNPs‐3 demonstrates overall superior performance compared to most existing counterparts when evaluated comprehensively in terms of both evaporation and thermoelectric properties (Table 1). While its thermoelectric performance is marginally lower than that of certain systems equipped with heat sink devices, it exhibits enhanced evaporation efficiency. Furthermore, the simpler system architecture significantly reduces construction complexity and improves operational convenience.

**TABLE 1:** Comparison of HSS@MNPs with other evaporators.

	Evaporation rate(kg m^−2^ h^−1^)	Output voltage(mV)	With heat sink(Yes/No)	Ambient temperature(°C)	Article acceptance date
**Ref. [** 34 **]**	2.02 kg m^−2^ h^−1^	107.4 mV	Yes	25°C	2023
**Ref. [** 35 **]**	1.75 kg m^−2^ h^−1^	261.4 mV	Yes	25°C	2025
**Ref. [** 36 **]**	2.83 kg m^−2^ h^−1^	58.0 mV	Yes	25°C	2025
**Ref. [** 37 **]**	1.72 kg m^−2^ h^−1^	108.4 mV	Yes	25°C	2025
**Ref. [** 38 **]**	0.99 kg m^−2^ h^−1^	90.7mV	No	22°C	2020
**Ref. [** 39 **]**	.0.84 kg m^−2^ h^−1^	100mV	Yes	——	2021
**Ref. [** 45 **]**	1.85 kg m^−2^ h^−1^	286.6 mV	No	25–28°C	2022
**Ref. [** 46 **]**	1.42 kg m^−2^ h^−1^	53.0 mV	No	——	2025
**Ref. [** 47 **]**	1.52 kg m^−2^ h^−1^	66.7 mV	No	25°C	2024
**Ref. [** 48 **]**	2.29 kg m^−2^ h^−1^	∼100 mV	Yes	——	2024
**Ref. [** 49 **]**	1.37 kg m^−2^ h^−1^	130 mV	No	——	2023
**Ref. [** 50 **]**	2.49 kg m^−2^ h^−1^	201 mV	Yes	20°C	2023
**This work**	3.08 kg m^−2^ h^−1^	140.6 mV	No	16°C	——

**FIGURE 7:**
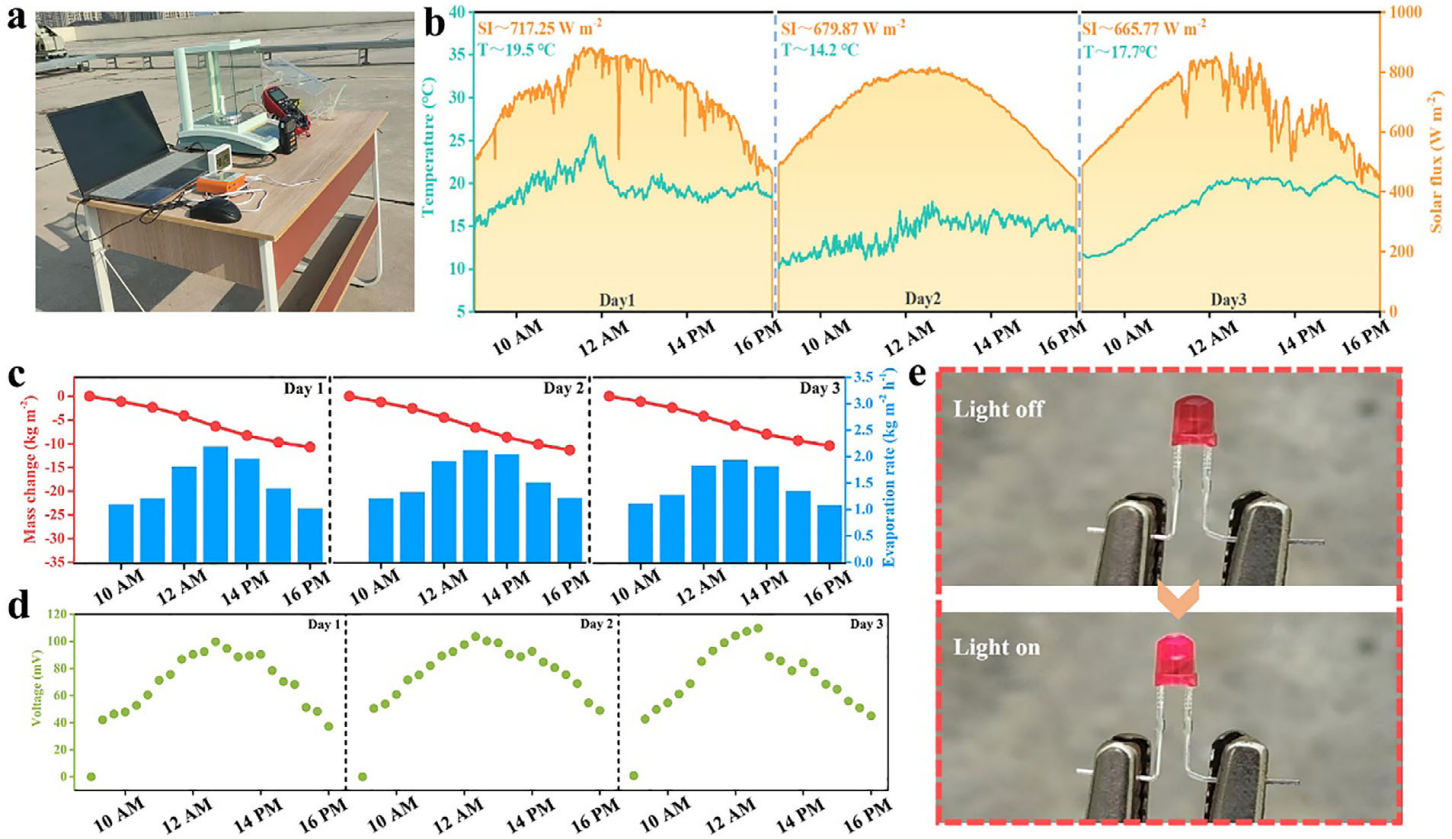
(a) Photograph of the outdoor testing apparatus in a real‐world setting. (b) Variation curves of light intensity and ambient temperature during testing. (c) Mass change of water. (d) Output voltage of TE during testing. (e) The LED before and after being illuminated by TE modules.

As a proof of concept, a simple test system was established to evaluate the evaporation‐thermoelectric co‐generation performance of the HSS@MNPs‐3 (the distance from the cold ends of the modules to the top of the HSS@MNPs‐3 is approximately 0.5 cm) under outdoor conditions (Figure 7a). A three‐day continuous experiment was conducted, with daily testing from 9 AM to 4 PM. This time frame was selected due to the strong solar irradiation, which provided suitable conditions for the water‐electricity co‐generation system. Throughout the test, key parameters including ambient temperature, light intensity, water quality variations, and module output voltage were recorded (Figure 7b). The results indicated that as light intensity increased, the water evaporation rate of the HSS@MNPs‐3 accelerated significantly, accompanied by a corresponding rise in output voltage. Over the three consecutive days, the average evaporation rates were 1.53, 1.62, and 1.49 kg m^−2^ h^−1^ Figure 7c, respectively, while the output voltage varied within the range of 37 to 103 mV Figure 7d. When multiple modules are connected in series, the generated electrical energy is sufficient to light up an LED lamp Figure 7e, demonstrating the material's favorable stability under real‐world application conditions. Considering the photothermal particles utilized in this study are derived from the ink sacs of edible squid—typically discarded as waste—while the organic silicon materials employed in the synthesis are common, low‐cost chemical reagents with straightforward preparation procedures. As a result, the overall production cost of this material remains relatively low. The preparation cost of the TE module‐containing material presented in this study is approximately US$2.4, which supports its feasibility for large‐scale production (Figure 7).

## 3 Conclusion

In summary, we successfully prepared a yolk@shell structured photothermal evaporator composed of super‐elastic hydrophobic silicone sponge, super‐hydrophilic epoxy resin and photothermal melanin nanoparticles via simple sol‐gel and spray coating methods. The results show that the prepared evaporator can effectively purify various waste liquids, such as acidic solution (pH∼*1*), alkaline solution (pH∼*13*), dye wastewater, heavy metal wastewater and 3.5 wt.% NaCl solution. Furthermore, a series of experiments and analyses have explored the influence of the evaporator height and the position of the thermoelectric (TE) module in the evaporator on the water evaporation performance and thermoelectric performance, and demonstrated that within a certain range, the height of the evaporator is positively correlated with evaporation performance, but it is not conducive to the improvement of thermoelectric performance. Ultimately, by taking advantage of the unique yolk@shell structure of the evaporator, the position of the TE module was optimized. A stable thermoelectric output of 135.4–144.6 mV and an evaporation rate of 3.08–3.17 kg m^−2^ h^−1^ were simultaneously achieved under one sun irradiation indoors, and stable evaporation‐thermoelectric cogeneration performance was also demonstrated in outdoor applications, offering a straightforward strategy to resolve the long‐standing trade‐off between high‐speed water evaporation and efficient thermoelectric generation.

## Conflicts of Interest

The authors declare no conflicts of interest.

## Supporting information




**Supporting File**: advs73926‐sup‐0001‐SuppMat.doc

## Data Availability

The data that support the findings of this study are available from the corresponding author upon reasonable request.
