# Supplementary material for: A Yolk@Shell Photothermal Structure for Integrated Solar‐Driven Undrinkable Water Purification and Thermoelectric Power Generation
Source: Adv Sci (Weinh). 2026 Jan 25;13(18):e23455. doi: 10.1002/advs.202523455 (PMC13042469; doi:10.1002/advs.202523455)
Supplement: Supplementary file 1 — Supporting File: advs73926‐sup‐0001‐SuppMat.doc [file ADVS-13-e23455-s001.doc]

**A Yolk@Shell Photothermal Structure for Integrated Solar-Driven Undrinkable Water Purification and Thermoelectric Power Generation**

Minrui Zhan^1#^, Xiang Fu^1#^, Yumei He^1^, Manman Zhao^1^, Xi Zhang^1^, Rong Liu^1^, Haonan Yang^1^, Ming Yang^3^, Huiyu Yang^2^*, Pei Lyu^1^, Jiehao Du^1^, Shaojin Gu^1^, Xin Liu^1^*, Bin Shang^1^*

^1^ State Key Laboratory of New Textile Materials and Advanced Processing, School of Materials Science and Engineering, Wuhan Textile University, Wuhan, 430200, P. R. China

^2^ School of Chemistry and Materials Science, Hubei Engineering University, Xiaogan 432000, China

^3^ Hubei Integrative Technology and Innovation Center for Advanced Fiberous Materials

^#^ These two authors contributed equally to this work

*Corresponding author, E-mail: [bshang@wtu.edu.cn](mailto:bshang@wtu.edu.cn); xinliu@wtu.edu.cn; hy-yang_wtu@hotmail.com

**Experimental section**

*Materials*

Epoxy resin was bought from Pengsheng Material Co., LTD (Kunshan, China). Tritont X-100, acetone, methyltrimethoxysilane (MTMS), tetraethoxysilane (TEOS), dimethyldimethoxysilane (DMDMS), acetic acid (HAc), dihexadecyl dimethyl ammonium bromide (DHDAB), Na_2_CO_3_, hydrochloric acid (HCl), sodium hydroxide (NaOH), ferric chloride (FeCl_3_·6H_2_O) and methyl blue were supplied by Aladdin Chemical Reagent Corp. Copper sulfate (CuSO_4_), nickel chloride (NiCl_2_), stannous chloride (SnCl_2_) and manganese chloride (MnCl_2_) were bought from Macklin Chemical Reagent Corp. Deionized water was used throughout the experiments.

*Synthesis of hydrophobic silicone sponge*

A weak acidic buffer solution contains DHDAB (38 g), Na_2_CO_3_ (0.04 g), HAc (40 uL) and water (80 mL) was prepared at first. Subsequently, certain amounts of TEOS (2.08 g), MTMS (14.3 g) and DMDMS (7.2 g) were added into the weak acidic buffer solution. The obtained mixture was transferred into a sealed glass tube, which was placed in an oven at 95 °C. After hydrolytic condensation of 12 h, the product was washed with water for 3 times to remove the unreacted substances, and then dried for use.

*Synthesis of melanin nanoparticles (MNPs)*

Generally, the fresh cuttlefish is first dissected to obtain the cuttlefish ink sac. Subsequently, the MNPs dispersion derived from the cuttlefish ink sac is centrifuged (10000 rpm, 10 min) and washed with water three times to remove impurities. Finally, the MNPs are thoroughly dried through vacuum freeze-drying at 40 ℃ for 48 h prior to use.

*Preparation of* *yolk@shell structured photothermal silicone sponge*

The yolk@shell structured photothermal silicone sponge was prepared via a simple spray coating method. Typically, 0.5 g of MNPs, 3.4 g of epoxy resin, 2 g of Triton^TM^ X-100, and 20 mL of acetone (15 mL) were fully mixed at first. Subsequently, the obtained mixture was sprayed onto the surface of pre-prepared silicone sponge while the pressure of the spray gun was adjusted to 4 MPa, and each surface was sprayed for approximately 20 s. After drying at 60 ^o^C for 5 h, the yolk@shell photothermal silicone sponge was successfully prepared.

*Characterizations*

Scanning electron microscopy (SEM) (SU-5000, Hitachi, Japan) and transmission electron microscopy (TEM, JEOL JEM-2100, Japan) were applied to observe the morphology of the samples. Fourier transform infrared spectra were analyzed via a transform infrared spectroscope (FTIR, EQUINX55, Brucher Crop, Germany). The water contact angles were measured on a Dataphysics OCA 20 contact angle system with 2 μL of deionized water as the indicator. The transmission and reflectance spectra of the samples were obtained using a UV/vis/NIR spectrophotometer (UV-3600Plus, Shimadzu, Japan). Infrared images and temperature changes of the samples were recorded by an infrared thermal imager (FLIR E54). Inductively Coupled Plasma Optical Emission Spectrometer (ICP-OES) (Agilent 5110, United States) was used to detect the concentration of ions before and after purification. The mechanical properties of the samples were investigated using a universal testing machine (Instron 5943, Country of origin USA). The distribution of water in the samples was observed through a three-dimensional (3D) microscope (RH-2000, HIROX, Japan). The evaporation enthalpy of water was tested by DSC analysis (TA instruments Discovery TGA/DSC).

*Solar driven vapor generation measurements*

A xenon light source (PL-XQ500W) equipped with a standard AM 1.5G solar spectrum optical filter was used to produce simulated solar irradiation (1 kW m^-2^). During the solar driven vapor generation measurements, the environmental humidity is controlled at around 50%, and the environmental temperature varies according to the test requirements (approximately 28 ^o^C or 16 ^o^C). In order to evaluate the water evaporation performance, an electronic balance (FA2104, 0.0001) was used to monitor the mass change of water, and the water evaporation rate (
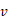
) could be calculated according to formula (1):


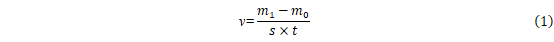


Here,
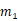
 and
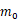
 denote the weight of water before and after light illumination, respectively. s represents the area of the tested samples, and t is the time of water evaporation.

*The proportion of energy in photothermal conversion process*

The energy input of the system mainly originates from Q_in_: HSS@MNPs solar energy absorbed


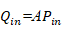
 (2)

Where *A* is the projected area (2.826×10^-3^ m^2^), *P_in_* is the intensity of sunlight per unit area (1000 W m^-2^).

The energy consumption of the system mainly originates from: conduction heat loss from HSS@MNPs to water; radiation heat loss from HSS@MNPs to the environment; convection heat loss from HSS@MNPs to the environment, and evaporative energy consumption of HSS@MNPs.

*Conduction heat loss η_cond_*

The conduction heat energy loss from HSS@MNPs-n to water is calculated as follows:


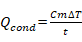
 (3)


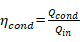
 (4)

Where *C* is the specific heat capacity of pure water (4.2 kJ kg^-1^ ℃^-1^), m denotes the weight of water (~ 25 g) in the test system, and *∆T* is increased temperature of the underlying bulk water, *t* is the irradiation time, *A* is the projected area (2.826×10^-3^ m^2^).

*Radiation heat loss η_rad_*

The radiation flux is based on Stefan-Boltzmann law, which is calculated as follows:


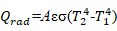
 (5)


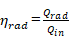
 (6)

Where
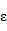
 (assumed to be 0.90) is the emissivity,
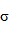
 is the Stefan-Boltzmann constant 5.67 ×10^-8^ W (m^2^ K^4^)^-1^, *T_2_* is the temperature at the surface of HSS@MNPs, and *T_1_* is the temperature of the adjacent environment of HSS@MNPs after irradiation, *A* is the projected area (2.826×10^-3^ m^2^).

*Convection heat loss η_conv_*

The convection heat loss is calculated based on Newton’s law of cooling:


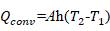
 (7)


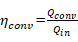
 (8)

Where *h* is convection heat transfer coefficient (assumed to be 5 W m^-2^ K^-1^); *T_2_* is the temperature at the surface of HSS@MNPs, and *T_1_* is the temperature of the adjacent environment of HSS@MNPs, *A* is the projected area (2.826×10^-3^ m^2^).

*Evaporative efficiency η_eva_*

The evaporative efficiency of HSS@MNPs is calculated as follows:


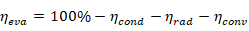
 (9)

Where *η_cond_*, *η_rad_*, and *η_conv_* denote the conduction, radiation, and convection heat loss components, respectively, of HSS@MNPs in photothermal conversion process.

The calculation results for the energy proportion in the photothermal conversion process of HSS@MNPs at different heights are as follows:

| HSS@MNPs | *ΔT* | *T_2_* | *T_1_* | *η_cond_* | *η_rad_* | *η_conv_* | *η_eva_* |
| --- | --- | --- | --- | --- | --- | --- | --- |
| HSS@MNPs-0.5 | 3.8 ℃ | 310.35 K | 304.25 K | 11.76% | 3.61% | 3.05% | 81.58% |
| HSS@MNPs-2 | 2.6 ℃ | 312.25 K | 306.35 K | 8.05% | 3.56% | 2.95% | 85.44% |
| HSS@MNPs-3 | 2.1 ℃ | 317.05 K | 310.35 K | 6.50% | 4.21% | 3.35% | 85.94% |
| HSS@MNPs-4 | 1.3 ℃ | 318.95 K | 310.55 K | 4.02% | 5.36% | 4.20% | 86.42% |

*The evaporative efficiency η_eva_ in photothermal conversion process*

The evaporative efficiency of HSS@MNPs-n is calculated as follows:


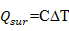
 (10)


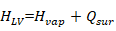
 (11)


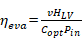
 (12)

Where *C* is the specific heat capacity of pure water (4.2 kJ kg^-1^ ℃^-1^), *∆T* is the surface temperature increase of HSS@MNPs during the photothermal evaporation process, *v* is the evaporation rate of HSS@MNPs, *H_vap_* is the evaporation enthalpy of HSS@MNPs (measured by DSC as 1445.73 J g^-1^), *C_opt_* is the optical concentration, and *P_in_* is the intensity of sunlight per unit area (1000 W m^-2^)

The calculation results for the evaporative efficiency in the photothermal conversion process of HSS@MNPs at different heights are as follows:

| HSS@MNPs | *ΔT* | *Q_sur_* | *H_LV_* | *v* | *η_eva_* |
| --- | --- | --- | --- | --- | --- |
| HSS@MNPs-0.5 | 9.1 ℃ | 38.22 J g^-1^ | 1483.95 J g^-1^ | 2.04 kg m^-2^ h^-1^ | 84.1% |
| HSS@MNPs-2 | 11.3 ℃ | 47.46 J g^-1^ | 1493.19 J g^-1^ | 2.41 kg m^-2^ h^-1^ | 99.96% |
| HSS@MNPs-3 | 15.7 ℃ | 65.94 J g^-1^ | 1511.67 J g^-1^ | 3.31 kg m^-2^ h^-1^ | 138.99% |
| HSS@MNPs-4 | 17.4 ℃ | 73.08 J g^-1^ | 1518.81 J g^-1^ | 3.14 kg m^-2^ h^-1^ | 132.47% |

*Solar driven thermoelectric generation measurements*

The commercial TE-module (TEP1-126T200, 4 cm × 4cm× 0.37 cm) was selected as the TE generation device to investigate the thermoelectric generation performance of different samples. All test samples are cylindrical in shape. Each has a diameter of approximately 6 cm, and their heights are approximately 0.5 cm, 2 cm, 3 cm, and 4 cm respectively. The TE module is embedded in the evaporator and its position inside the evaporator is adjusted as required. All experiments were conducted in an environment with 50% relative humidity, but the ambient temperature was respectively controlled at approximately 16 ^o^C, 20 ^o^C, and 28 ^o^C. A multimeter (FLUKE53-IIB) was used to measure the output voltage.


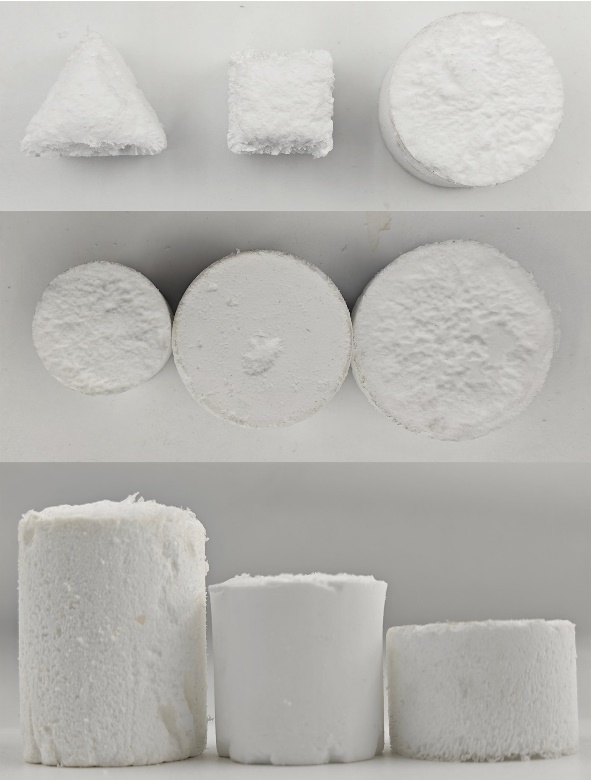


**Figure S1.** Photos of the prepared HSS of different shapes, sizes and heights.


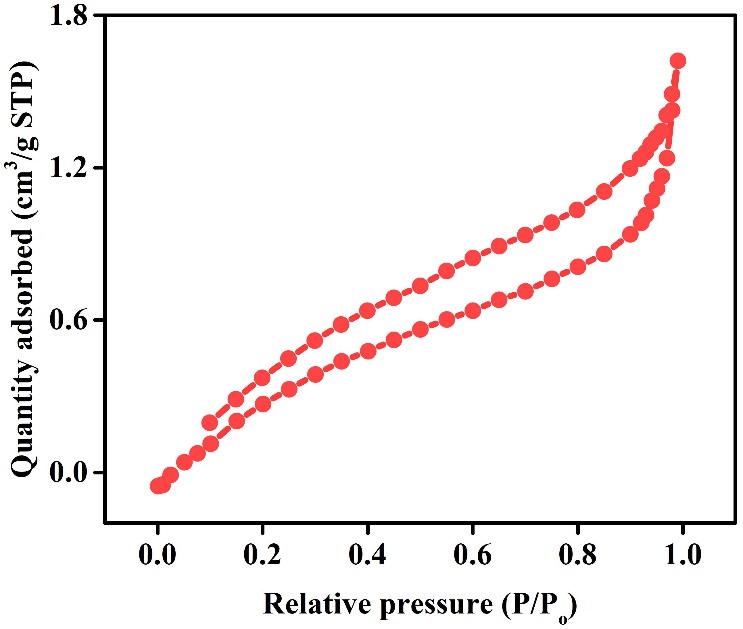


**Figure S2.** N_2_ adsorption-desorption isotherms of the prepared HSS.


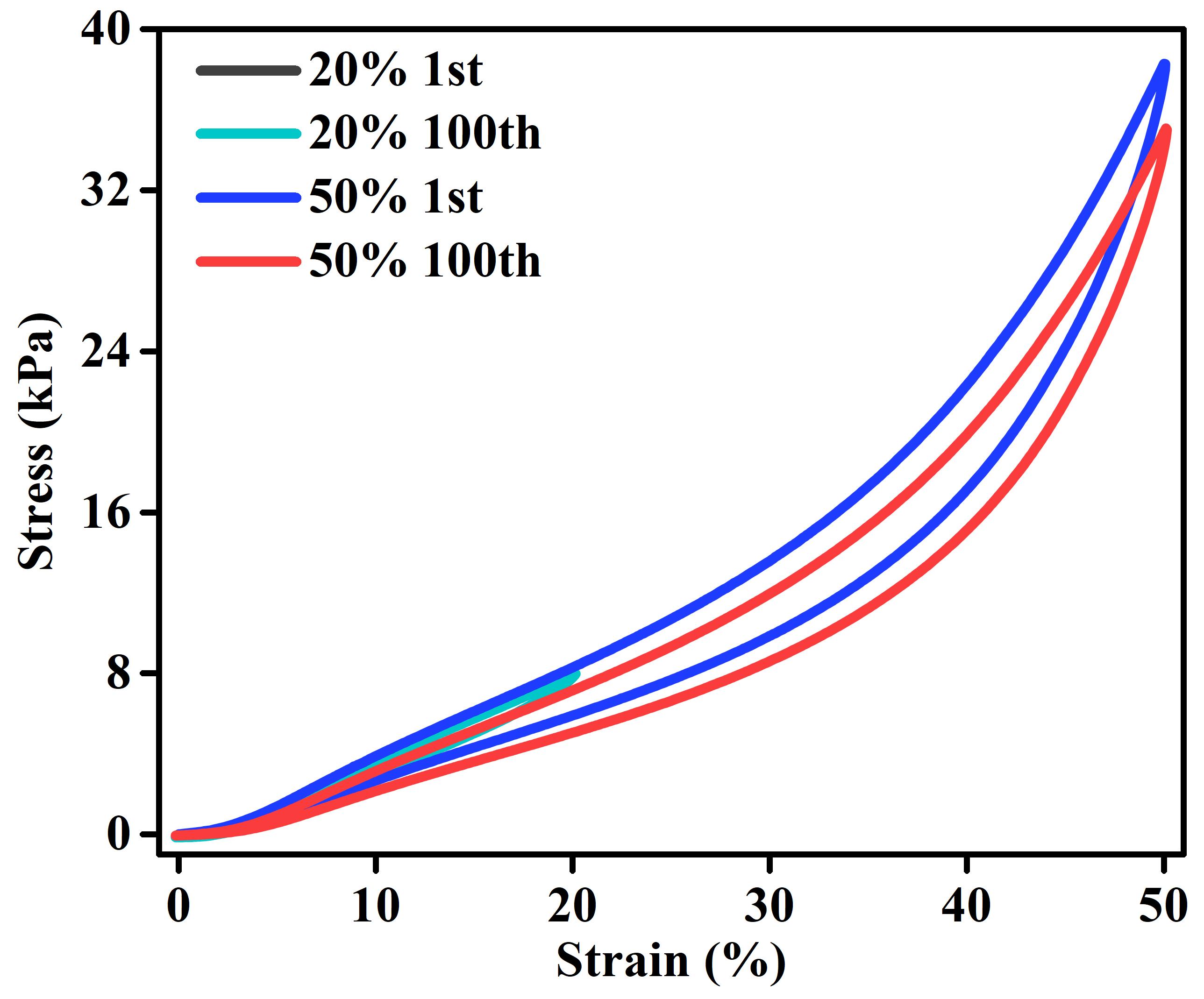


**Figure S3.** The stress-strain relationship curve of the prepared HSS under compression.


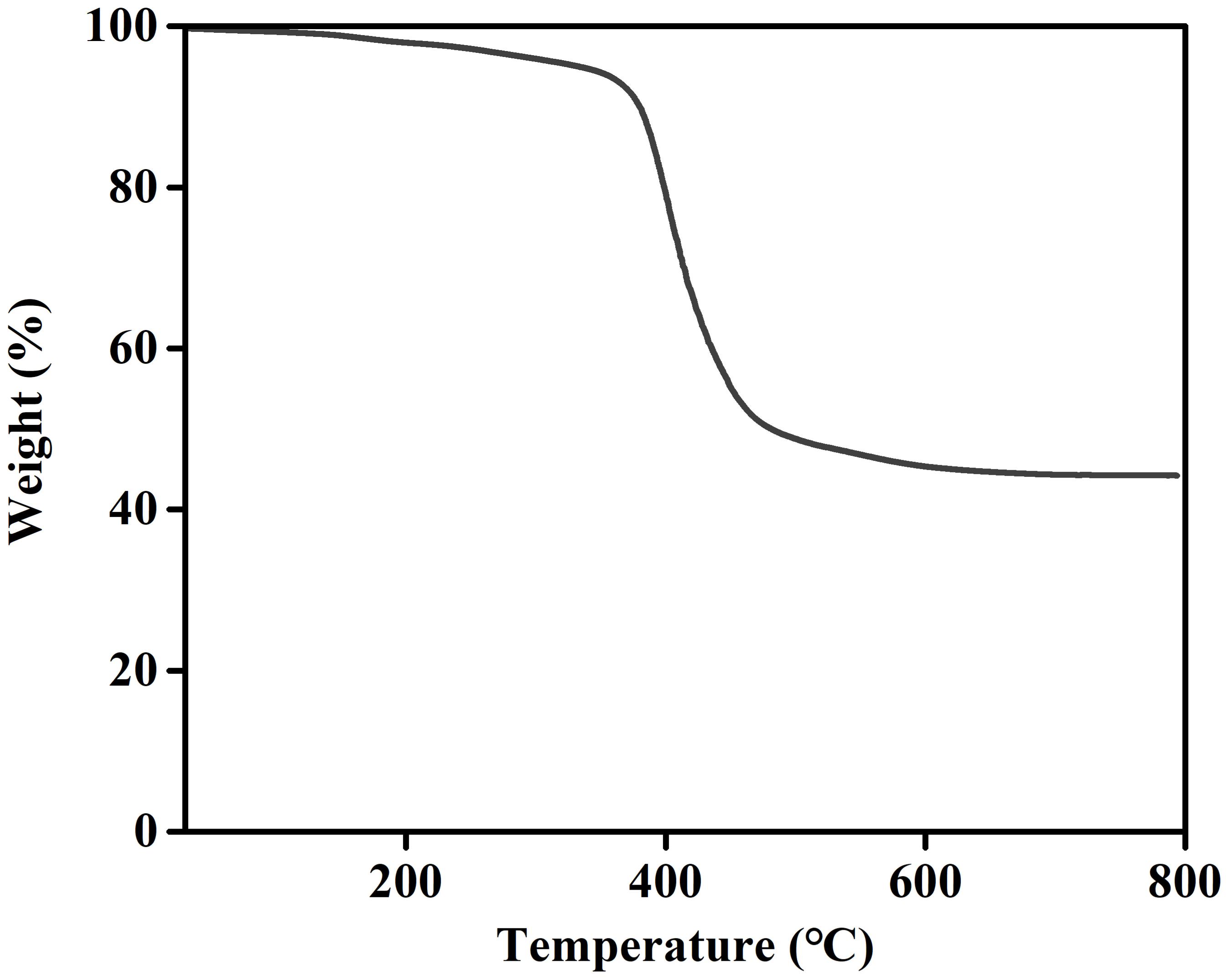


**Figure S4.** Thermogravimetric analysis curve of the prepared HSS.


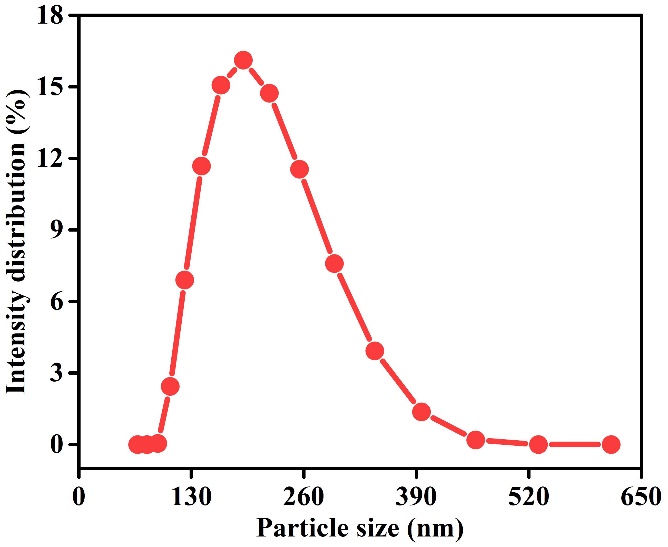


**Figure S5.** The particle size distribution of melanin particles.


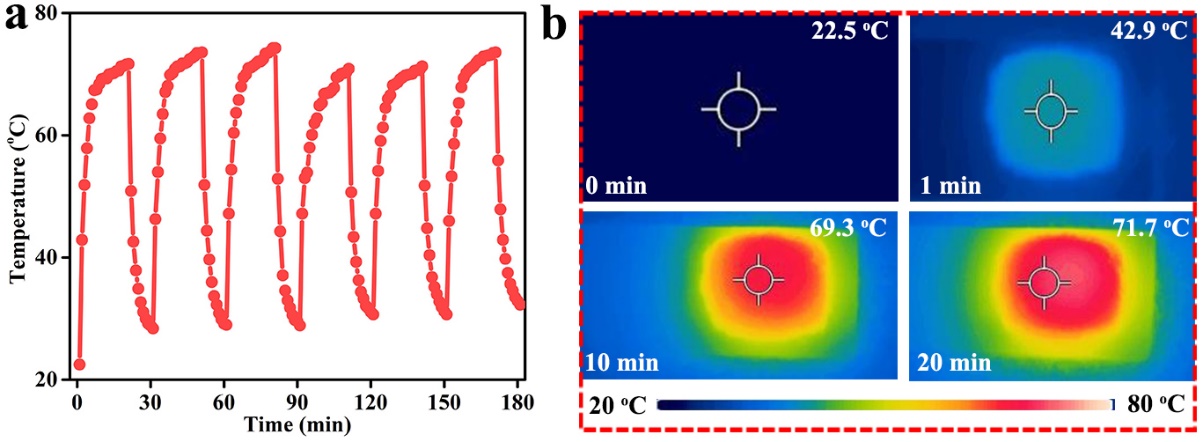


**Figure S6.** Surface temperature variation curve (a) and corresponding infrared images of the obtained melanin particles.


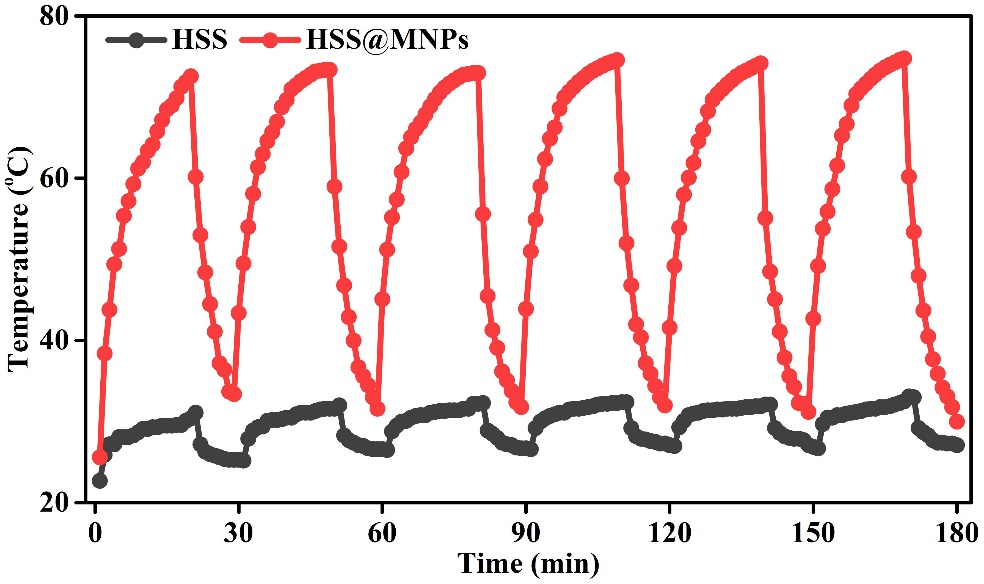


**Figure S7.** Surface temperature variation curve of the fabricated HSS and HSS@MNPs during in multiple cycle tests.


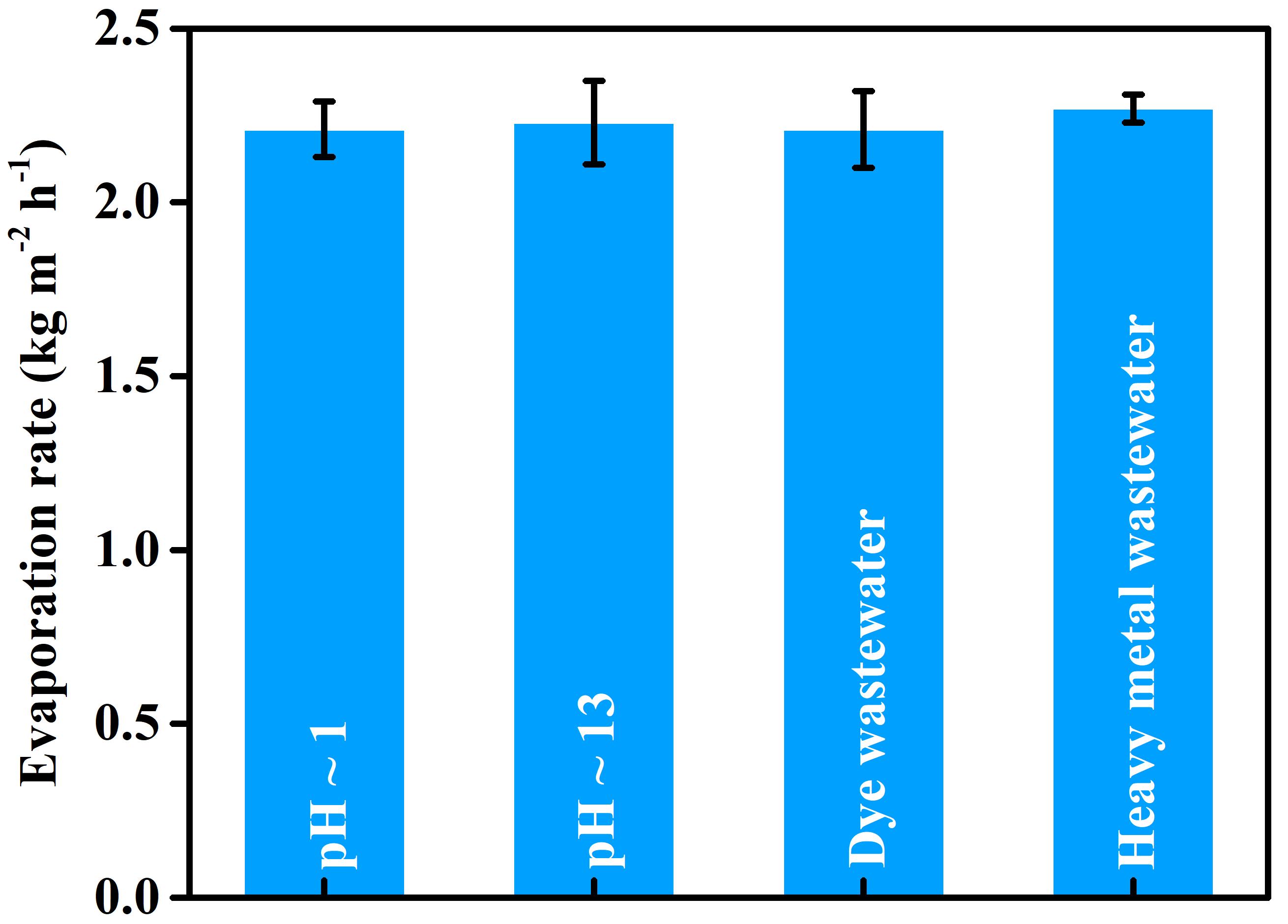


**Figure S8.** The water evaporation rate of HSS@MNPs when treating different types of wastewaters.


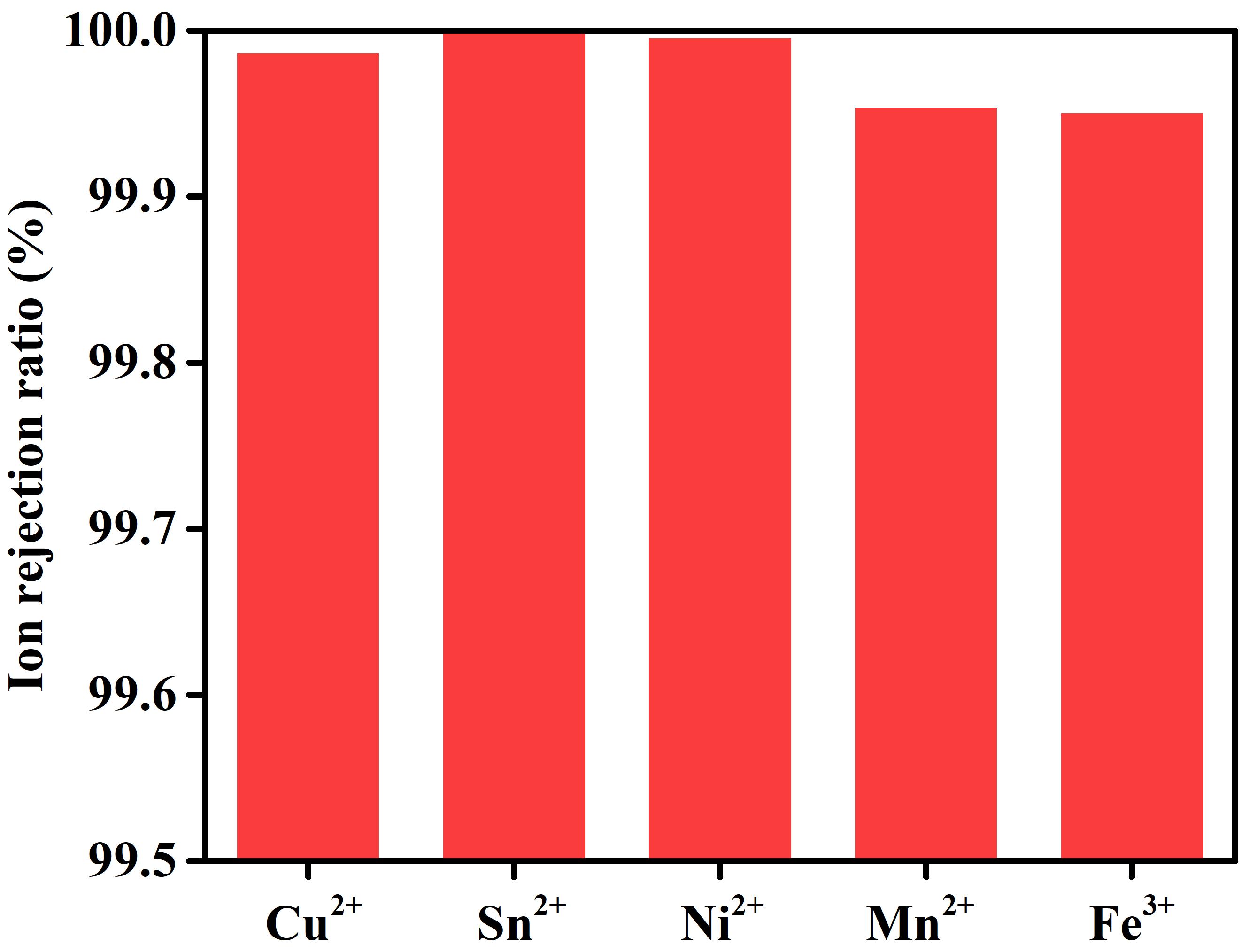


**Figure S9.** The rejection ratio of ions in heavy metal wastewater


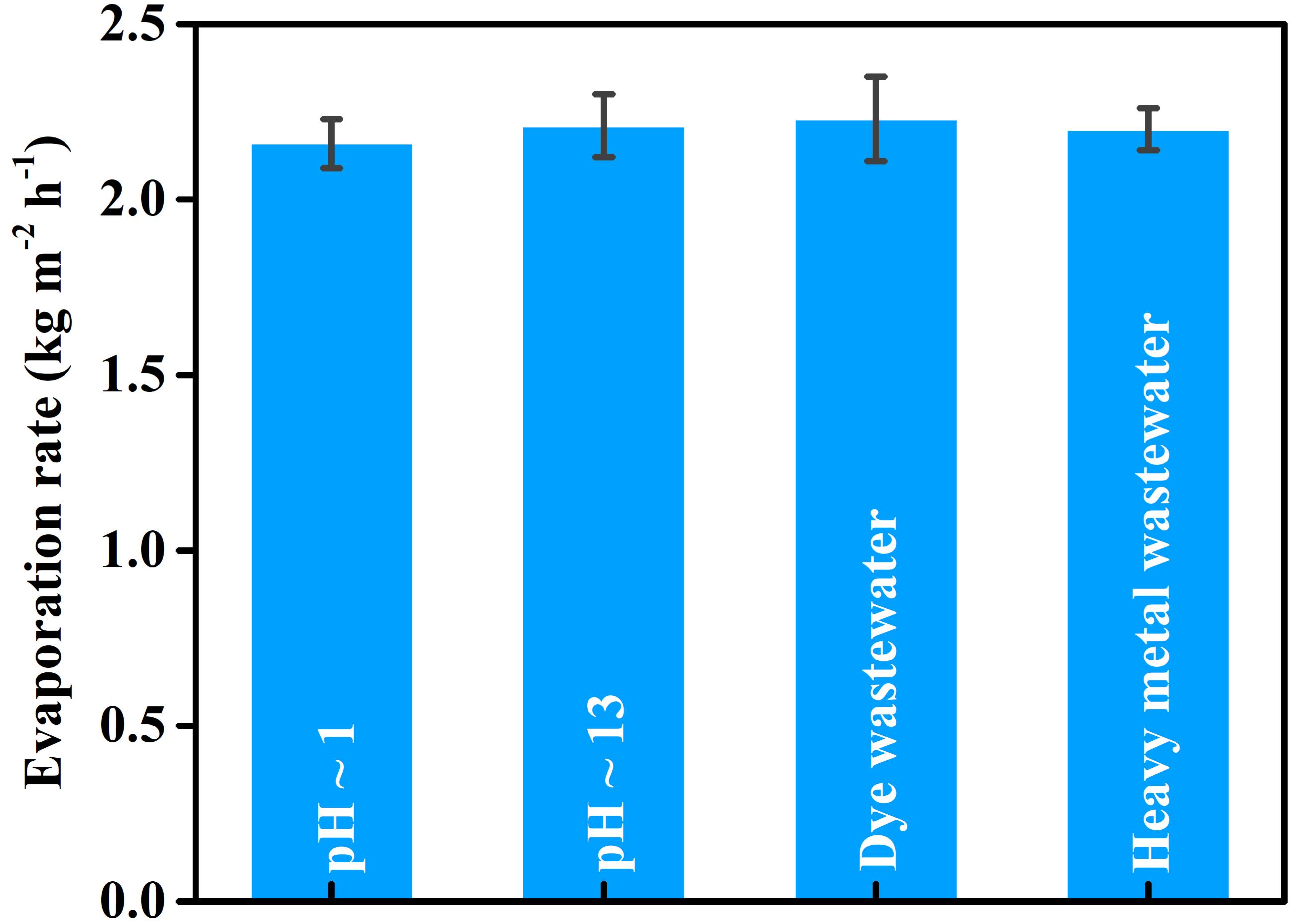


**Figure S10.** The water evaporation rate of HSS@MNPs after three days of continuous immersion in the waste liquids.


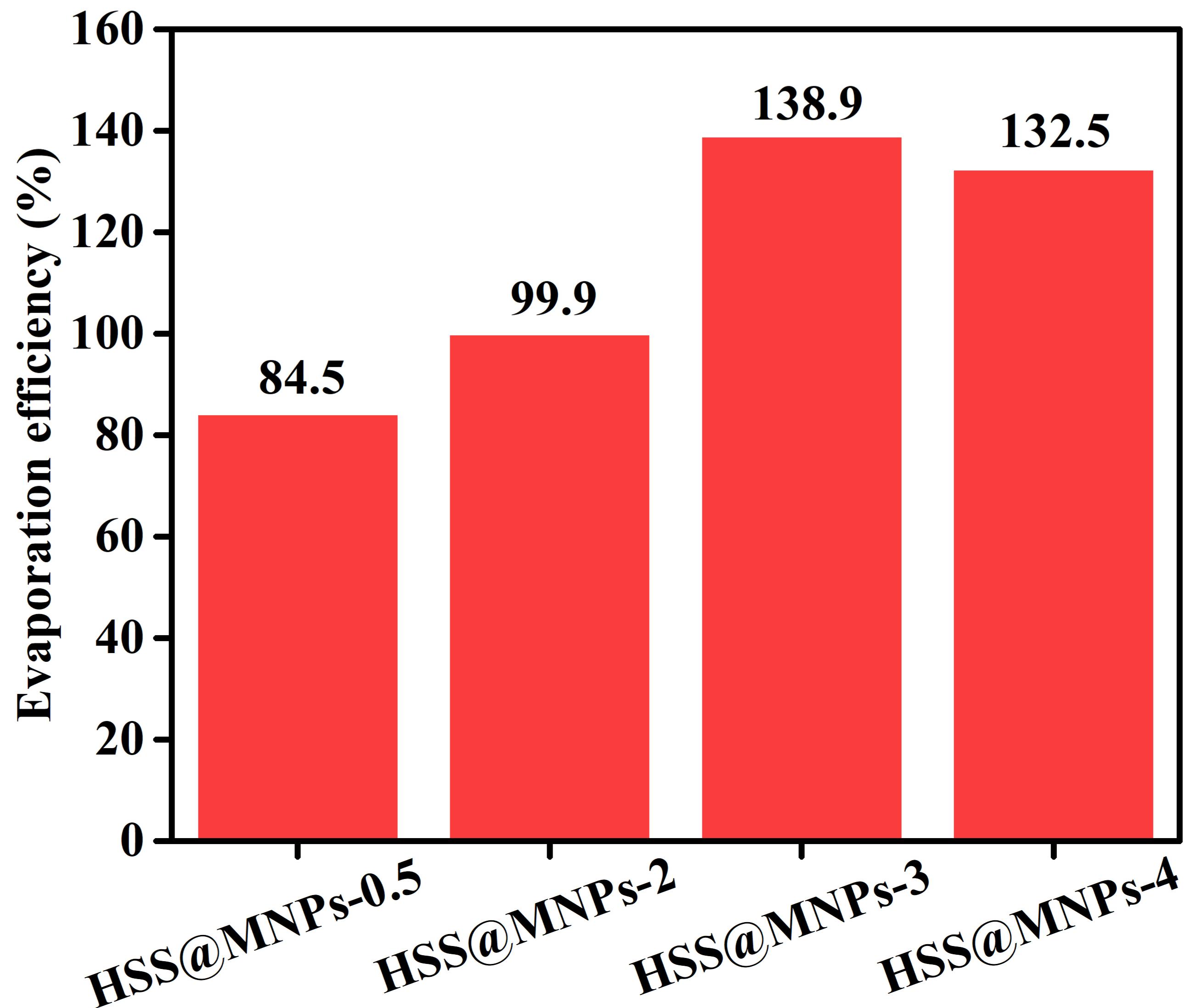


**Figure S11.** Evaporation efficiency of different evaporators.


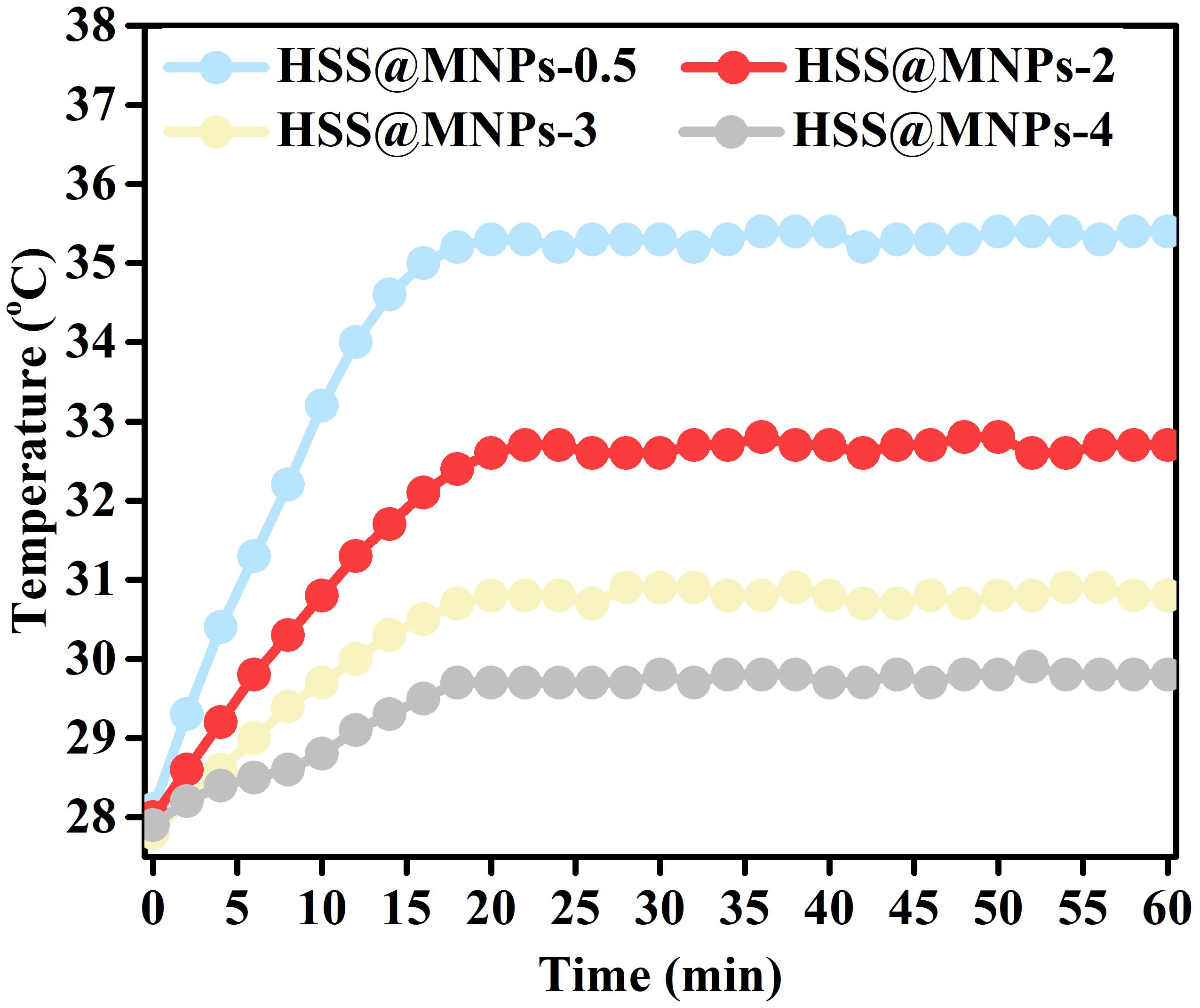


**Figure S12.** The temperature at the hot end of the module.


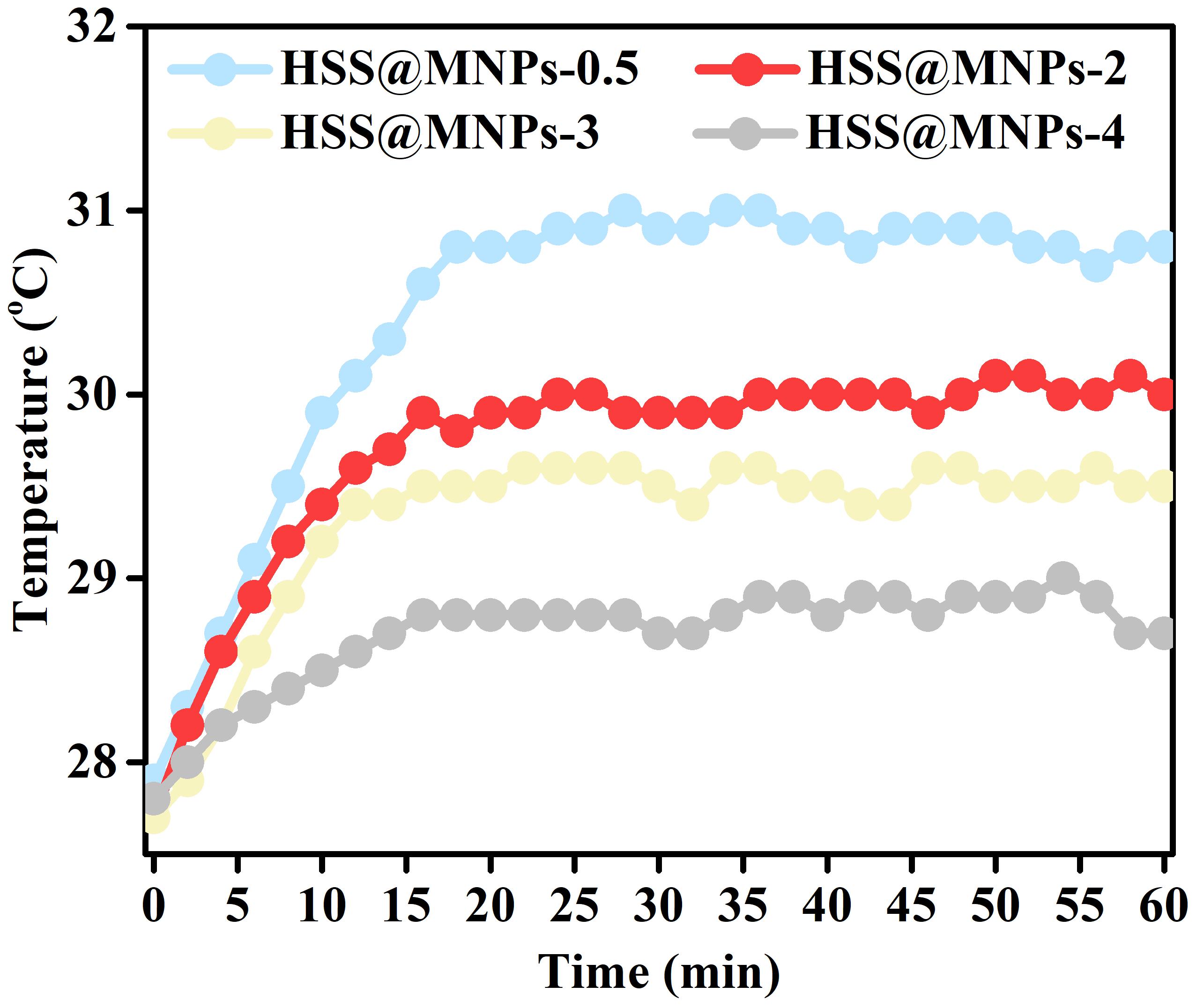


**Figure S13.** The temperature at the cold end of the module.

**
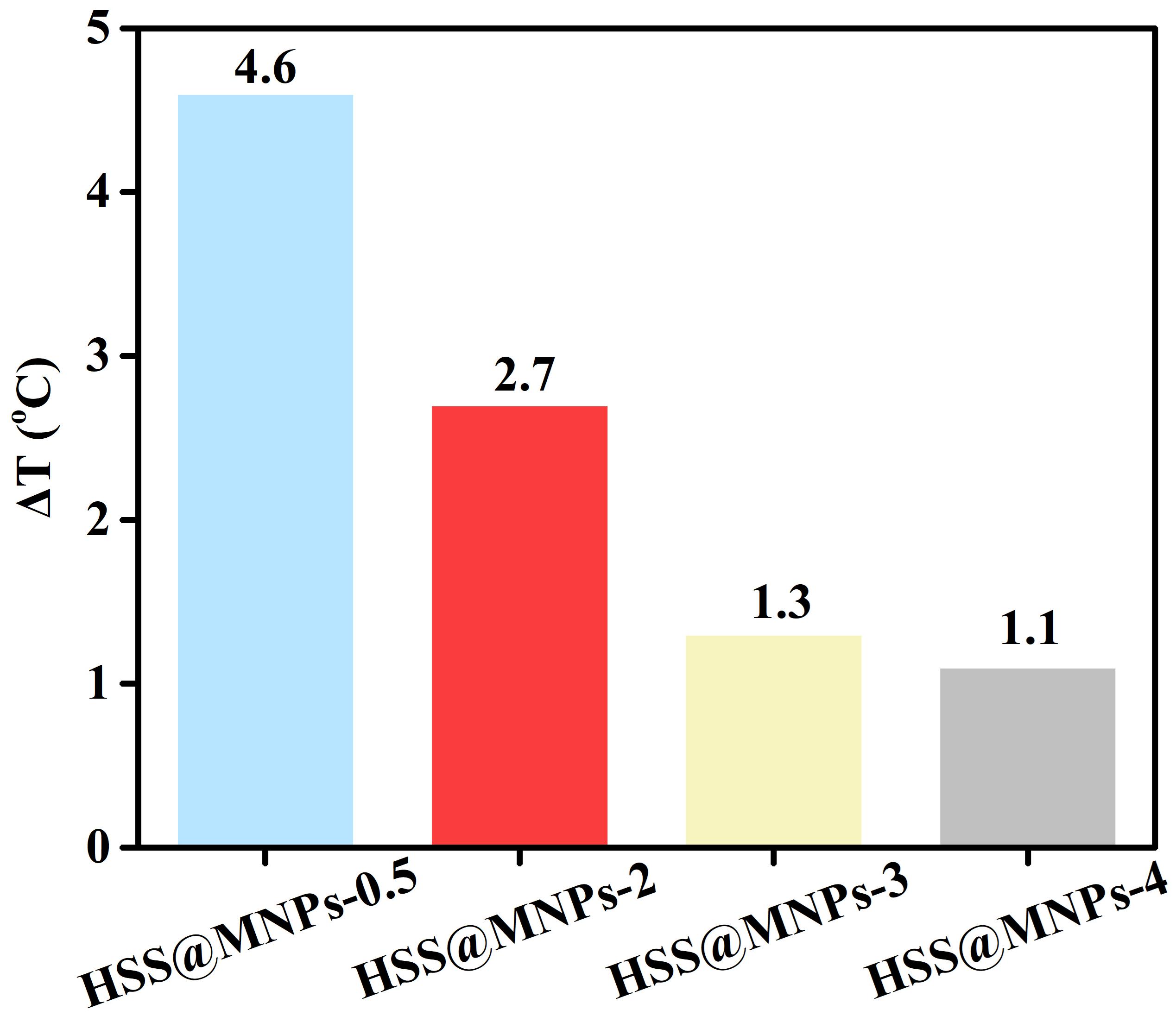
**

**Figure S14.** The temperature difference between the two ends of the module.


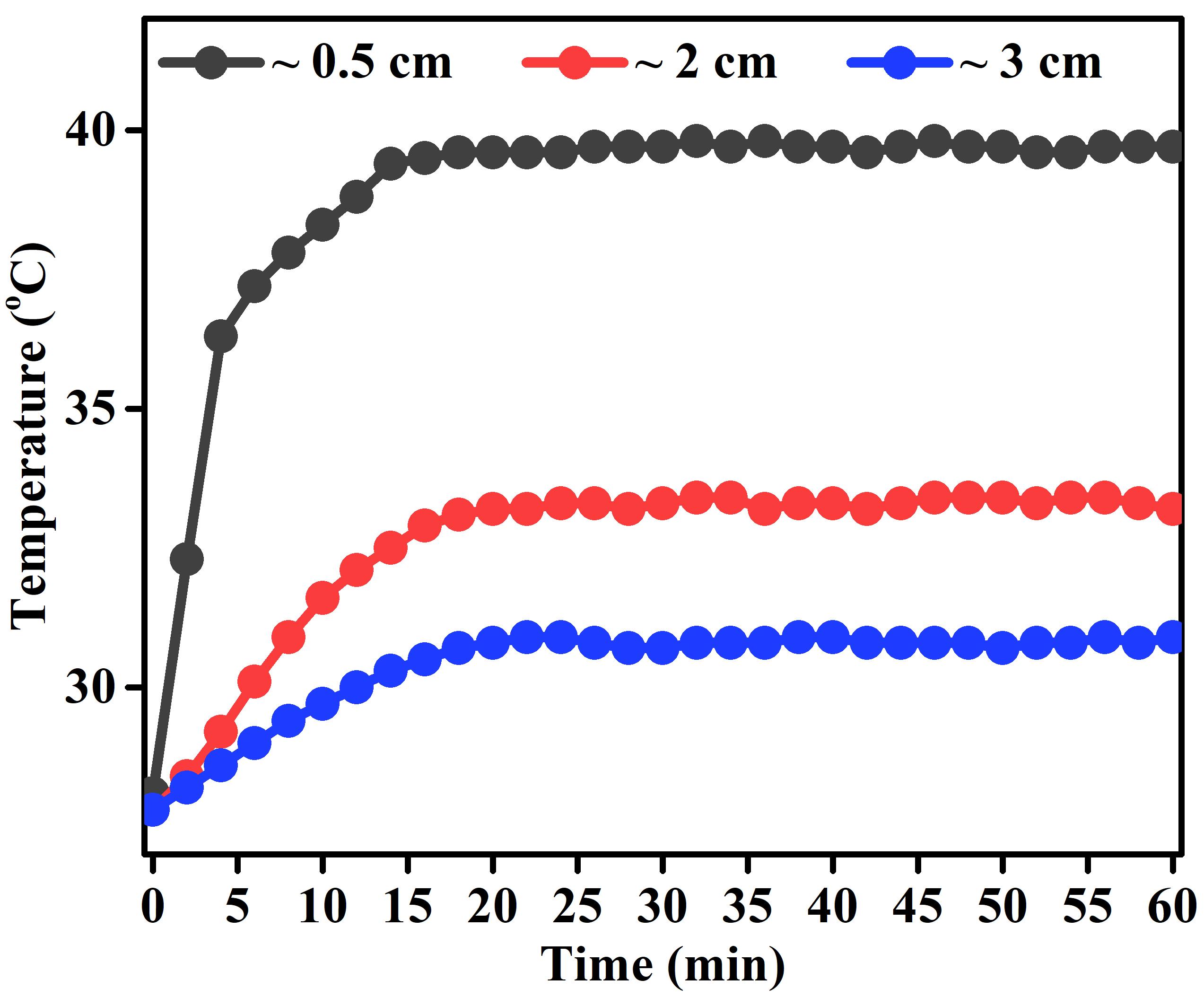


**Figure S15.** The temperature change of the hot end of the module when the module is placed at different positions in the HSS@MNPs-3.


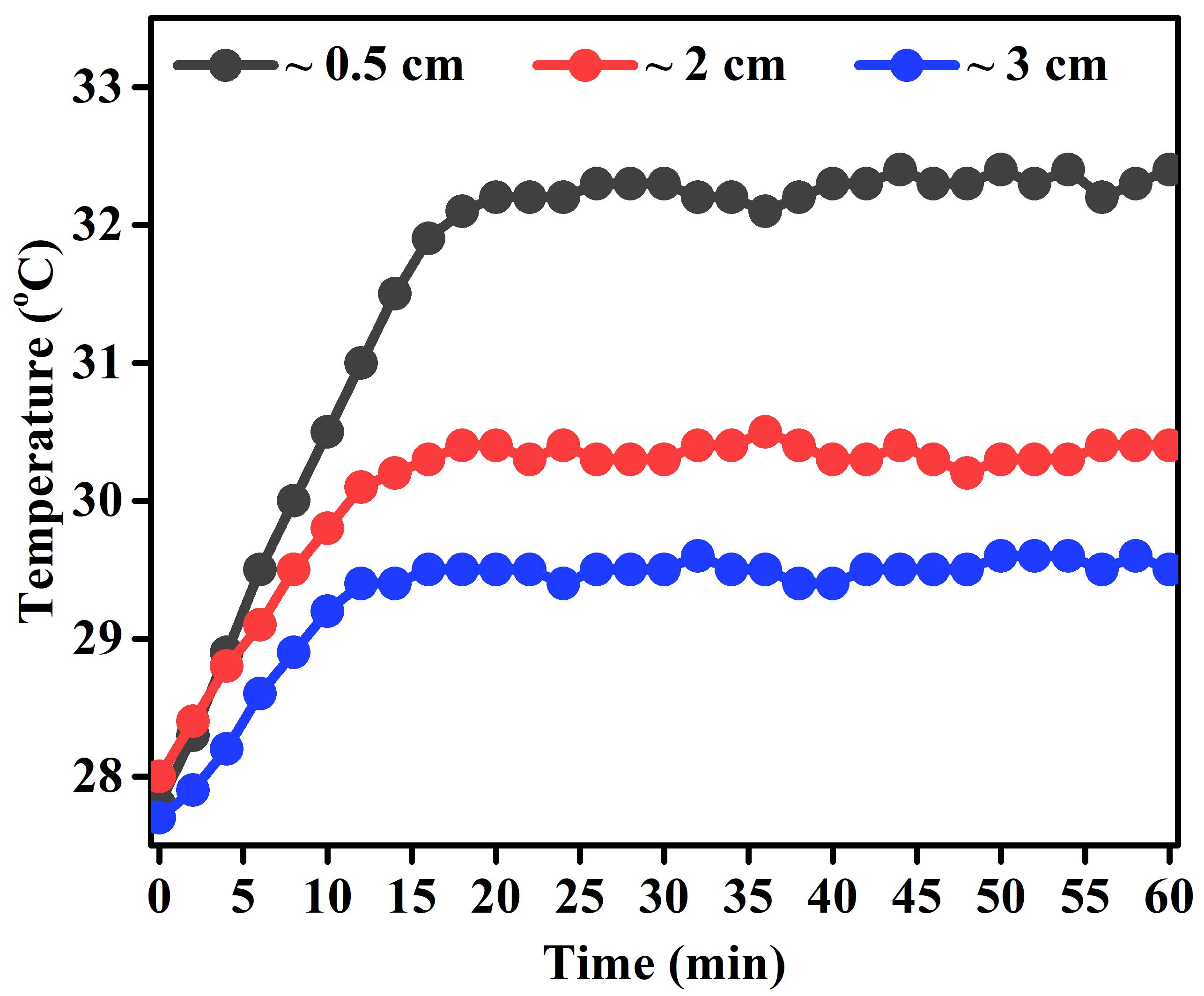


**Figure S16.** The temperature change of the cold end of the module when the module is placed at different positions in the HSS@MNPs-3.


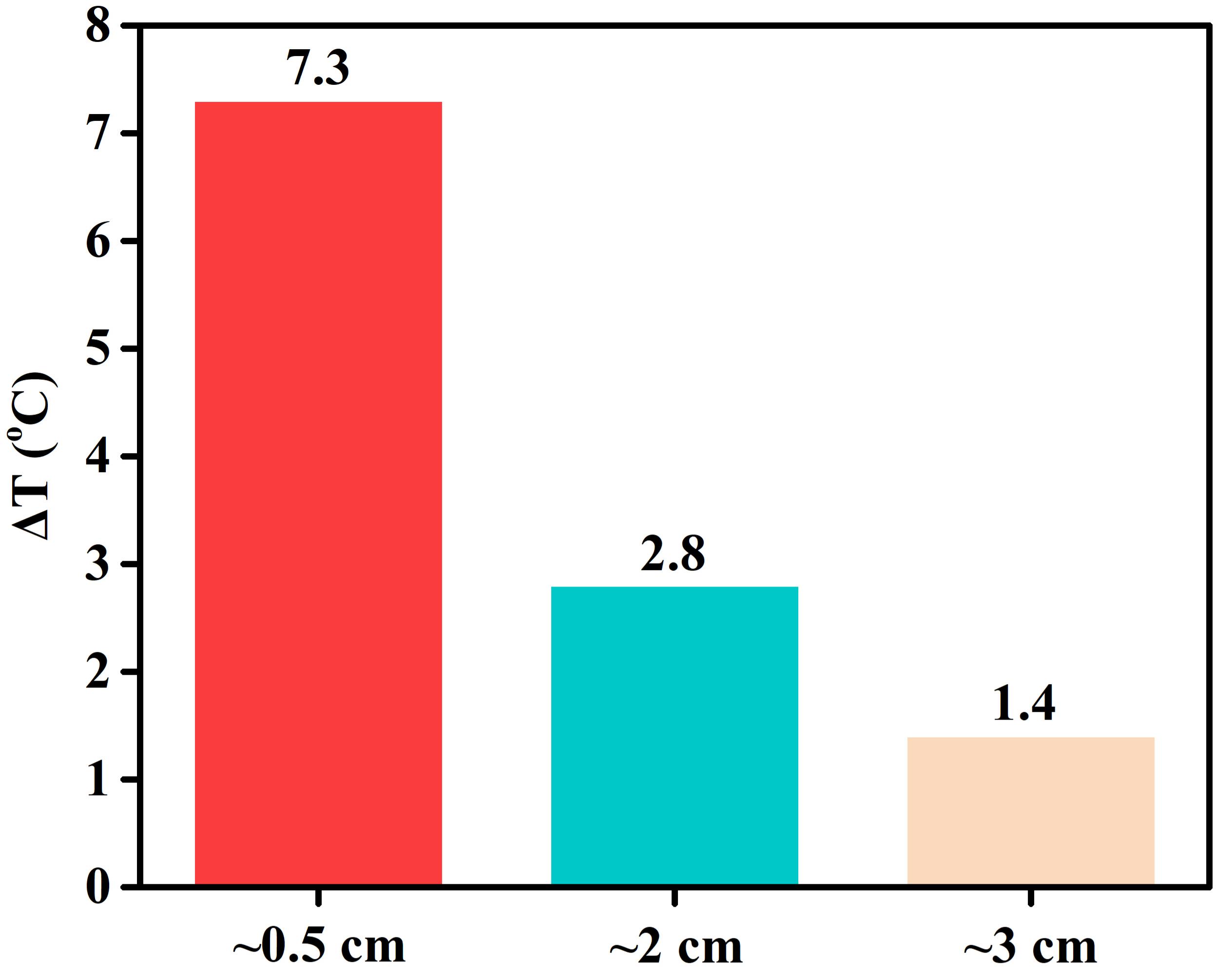


**Figure S17.** The temperature difference between the two ends of the module when the module is placed at different positions in the HSS@MNPs-3.


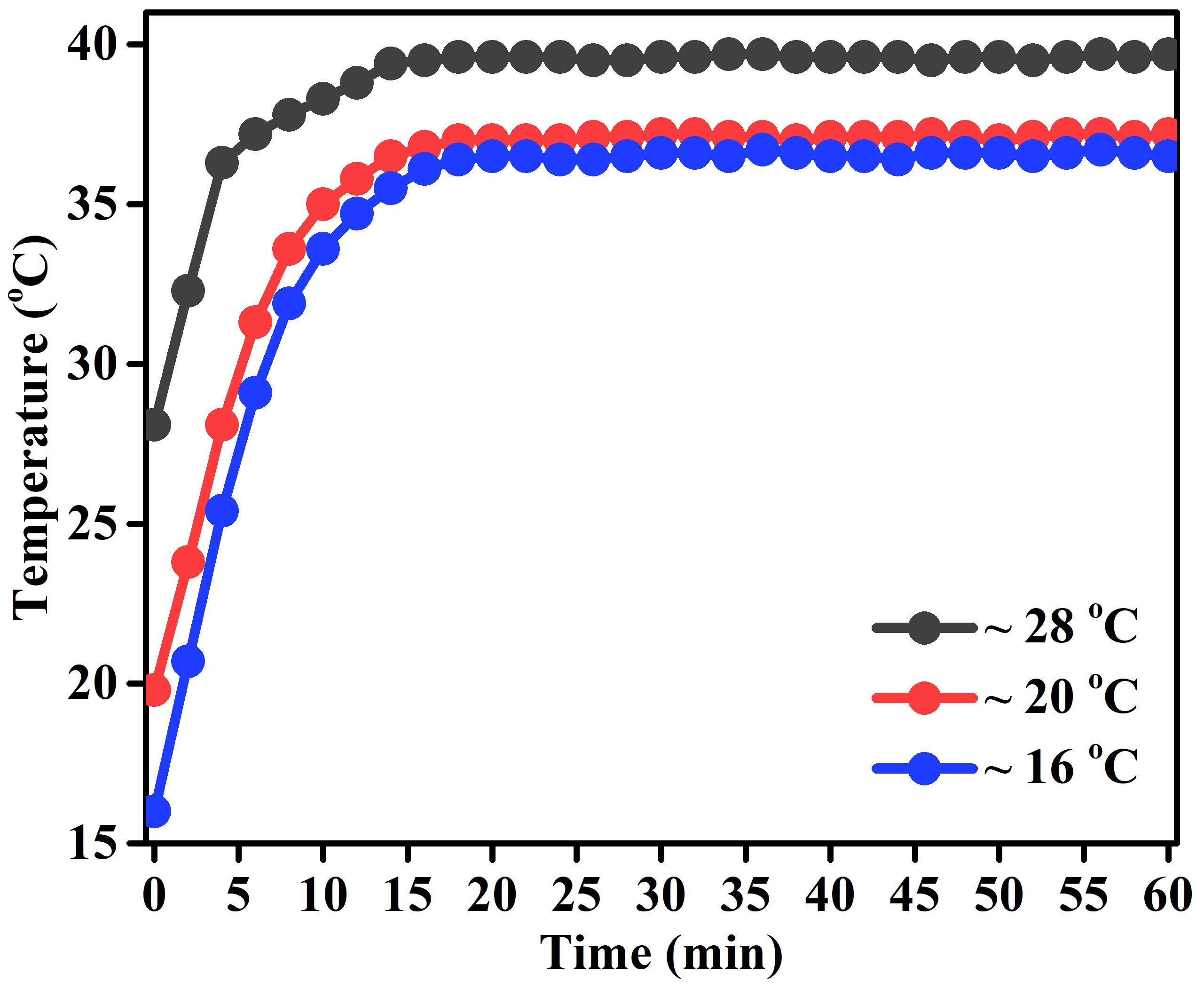


**Figure S18.** The temperature change of the hot end of the module when the cold end of the module is approximately 0.5 cm away from the top of the HSS@MNPs-3 under different test environments.


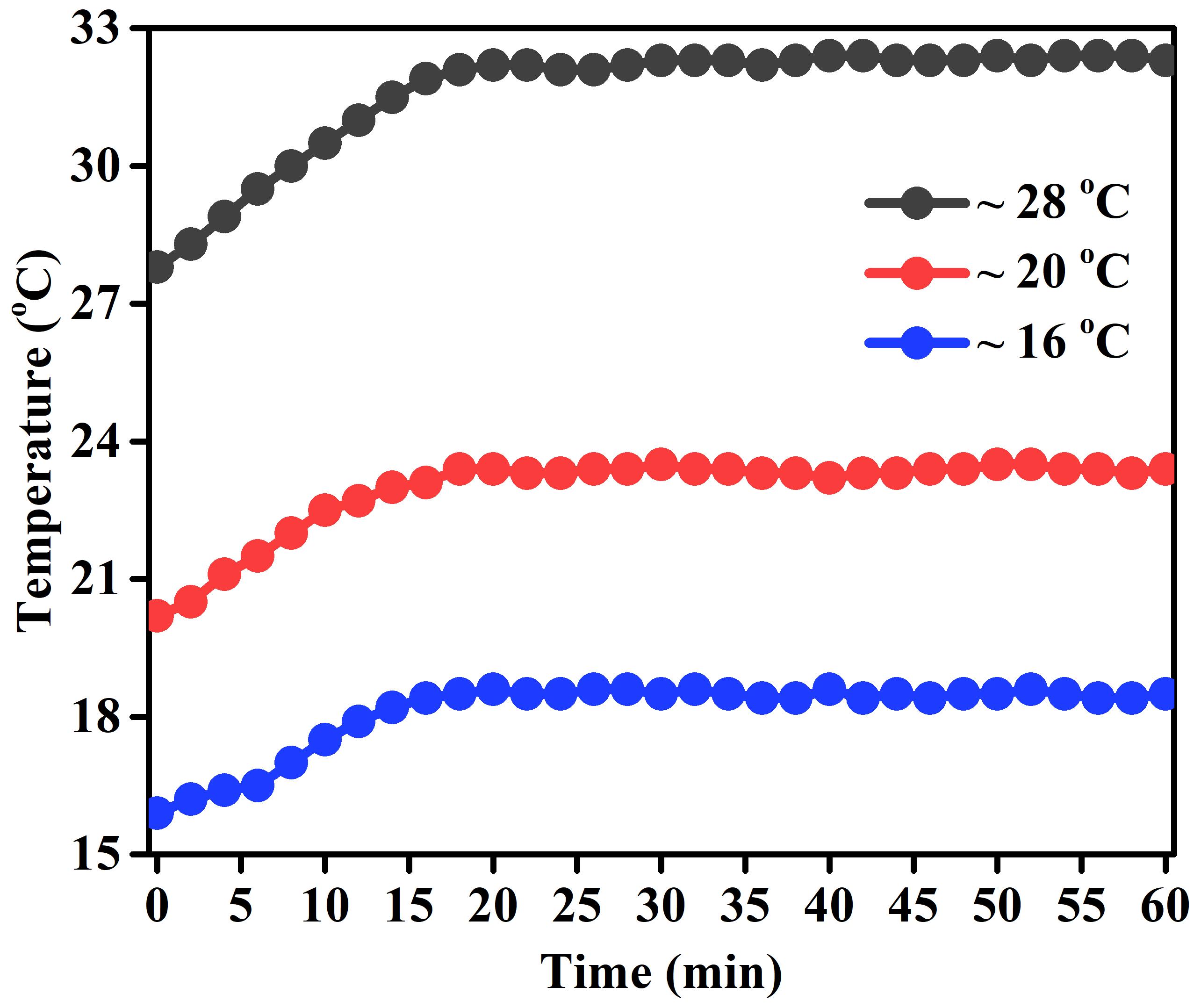


**Figure S19.** The temperature change of the cold end of the module when the cold end of the module is approximately 0.5 cm away from the top of the HSS@MNPs-3 under different test environments.


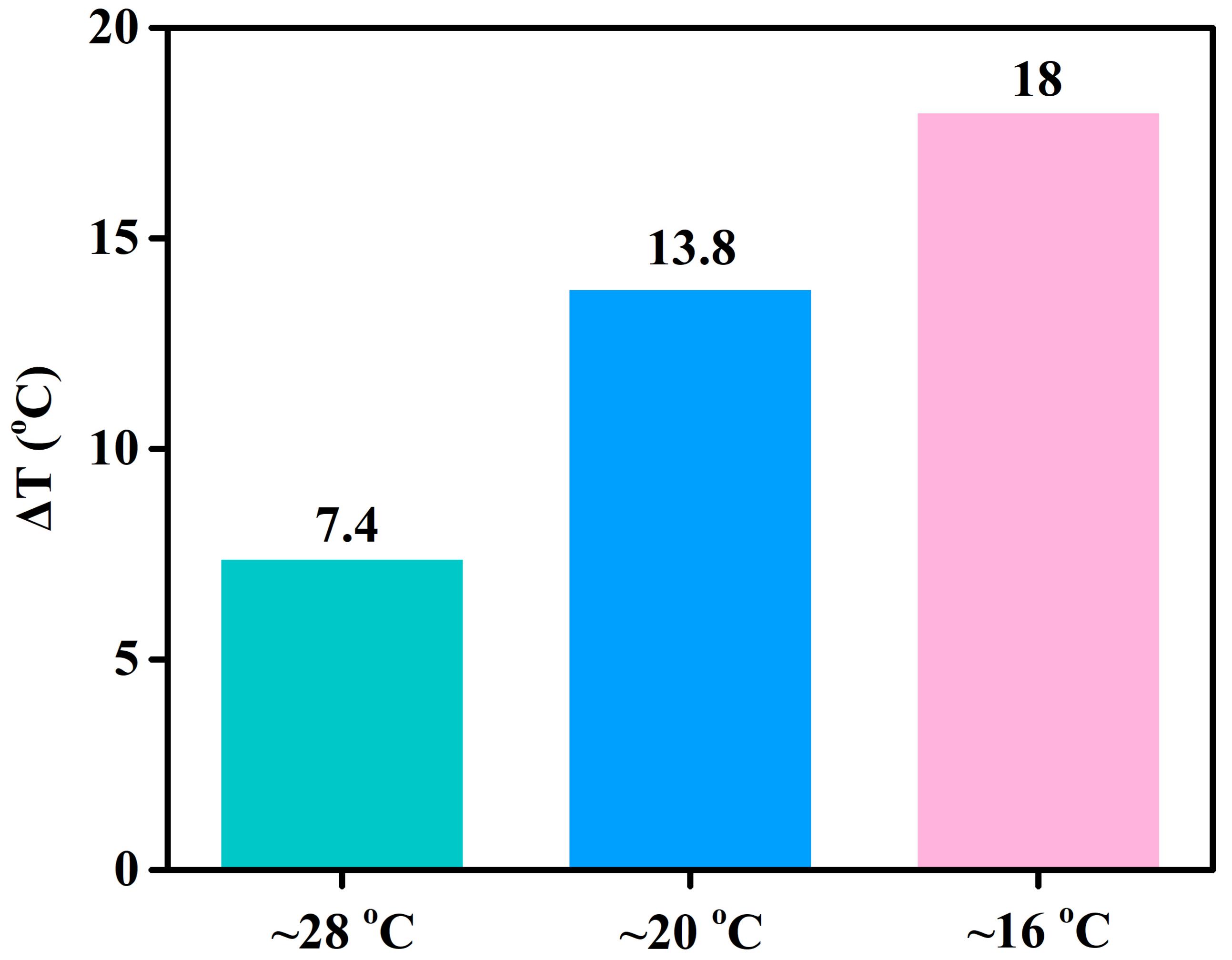


**Figure S20.** At different ambient temperature, the temperature difference between the two ends of the module.


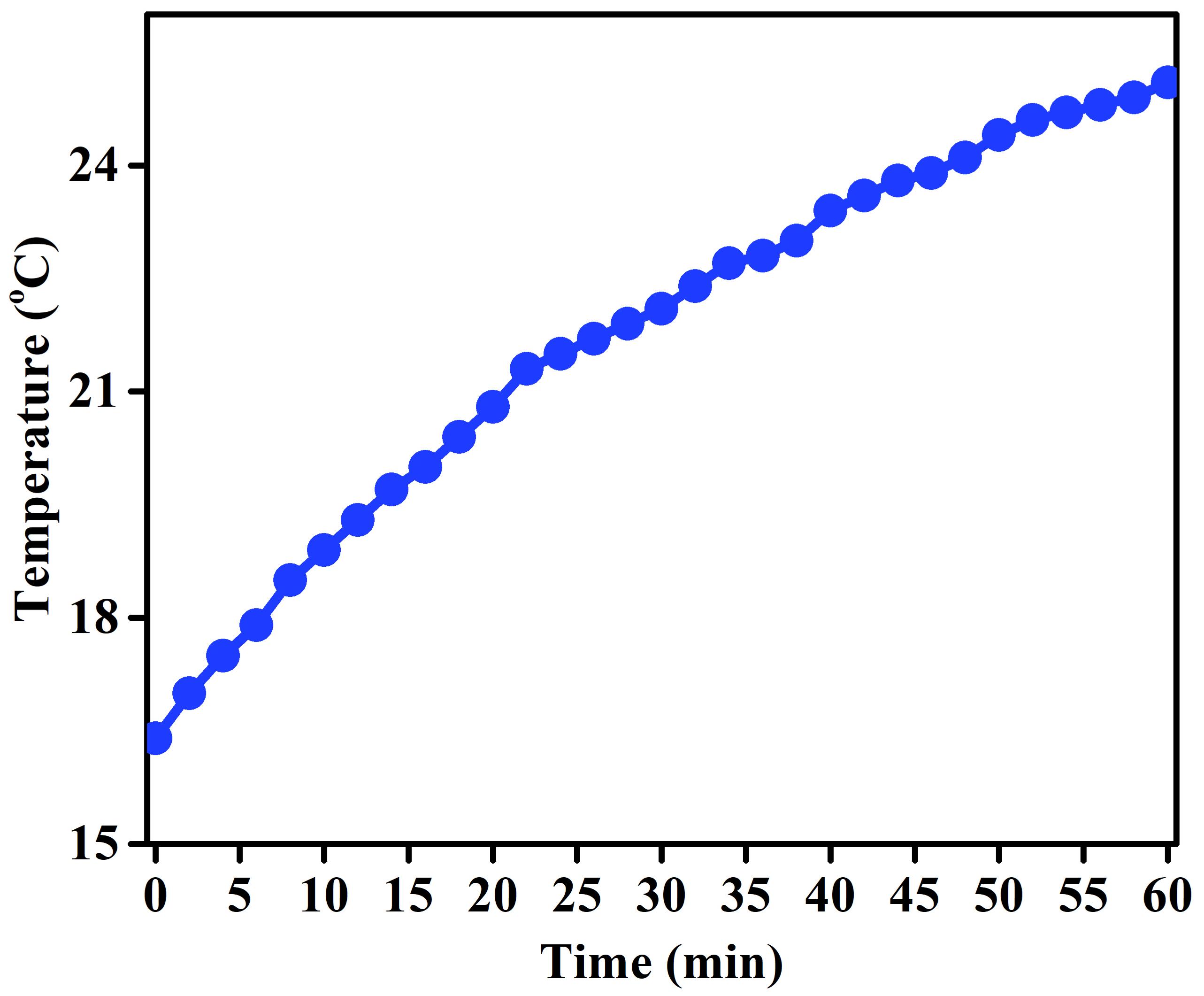


**Figure S21.** The temperature change of the cold end of the module when the module is directly inserted into the evaporator at the same height but retains its "core" structure below (the ambient temperature is approximately 16 ^o^C, and the module is approximately 0.5 cm away from the top of the HSS@MNPs-3).


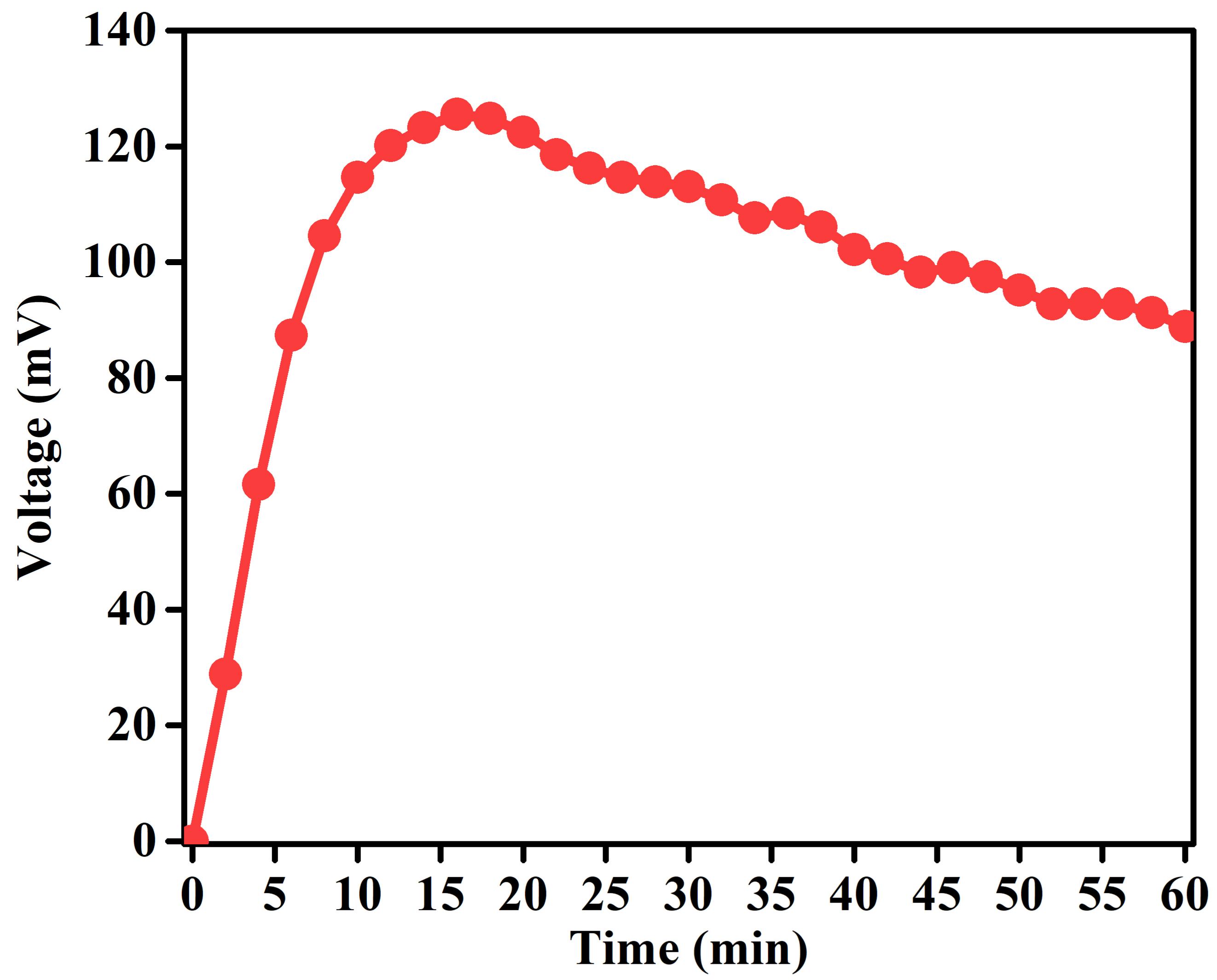


**Figure S22.** Voltage curves generated by the module when the module is directly inserted into the evaporator at the same height but retains its "core" structure below (the ambient temperature is approximately 16 ^o^C, and the module is approximately 0.5 cm away from the top of the HSS@MNPs-3).
